# Conditionally immortalised leukaemia initiating cells co-expressing *Hoxa9/Meis1* demonstrate microenvironmental adaptation properties ex vivo while maintaining myelomonocytic memory

**DOI:** 10.1038/s41598-021-84468-3

**Published:** 2021-03-05

**Authors:** Maike Stahlhut, Teng Cheong Ha, Ekaterina Takmakova, Michael A. Morgan, Adrian Schwarzer, Dirk Schaudien, Matthias Eder, Axel Schambach, Olga S. Kustikova

**Affiliations:** 1 Institute of Experimental Hematology, Hannover Medical School, Carl-Neuberg-Strasse 1, 30625 Hannover, Germany; 2 REBIRTH - Research Center for Translational Regenerative Medicine, Hannover Medical School, Hannover, Germany; 3 Department of Hematology, Hemostasis, Oncology and Stem Cell Transplantation, Hannover Medical School, Hannover, Germany; 4 Fraunhofer Institute for Toxicology and Experimental Medicine ITEM, Hannover, Germany; 5 Division of Hematology/Oncology, Boston Children’s Hospital, Harvard Medical School, Boston, MA USA

**Keywords:** Cancer, Cancer stem cells

## Abstract

Regulation of haematopoietic stem cell fate through conditional gene expression could improve understanding of healthy haematopoietic and leukaemia initiating cell (LIC) biology. We established conditionally immortalised myeloid progenitor cell lines co-expressing constitutive *Hoxa9.EGFP* and inducible *Meis1.dTomato* (H9M-ciMP) to study growth behaviour, immunophenotype and morphology under different cytokine/microenvironmental conditions ex vivo upon doxycycline (DOX) induction or removal. The vector design and drug-dependent selection approach identified new retroviral insertion (RVI) sites that potentially collaborate with *Meis1*/*Hoxa9* and define H9M-ciMP fate*.* For most cell lines, myelomonocytic conditions supported reversible H9M-ciMP differentiation into neutrophils and macrophages with DOX-dependent modulation of *Hoxa9/Meis1* and CD11b/Gr-1 expression. Here, up-regulation of *Meis1*/*Hoxa9* promoted reconstitution of exponential expansion of immature H9M-ciMPs after DOX reapplication. Stem cell maintaining conditions supported selective H9M-ciMP exponential growth. H9M-ciMPs that had *Ninj2* RVI and were cultured under myelomonocytic or stem cell maintaining conditions revealed the development of DOX-dependent acute myeloid leukaemia in a murine transplantation model. Transcriptional dysregulation of *Ninj2* and distal genes surrounding RVI (*Rad52, Kdm5a*) was detected. All studied H9M-ciMPs demonstrated adaptation to T-lymphoid microenvironmental conditions while maintaining immature myelomonocytic features. Thus, the established system is relevant to leukaemia and stem cell biology.

## Introduction

Genetic modification of the haematopoietic stem cells (HSC) via tetracycline-regulated gene transfer allows inducible and/or reversible expression of growth-controlling factors. The controllability of gene expression is essential since over-expressed transcription factors (TF) can trigger side effects resulting in cell exhaustion or malignant transformation, irreversible growth arrest or cell death due to apoptosis. Improved lentiviral doxycycline (DOX) controlled systems expressing single or multiple TFs could be useful to study both the benign and malignant expansion of genetically modified HSCs and open new routes in reprogramming, stem cell, and leukaemia biology^1,2^. However, tetracycline-regulated lentiviral vector efficiencies are often limited when in vivo transplantation models are used^3^.

Thus, a better understanding of intrinsic and extrinsic components influencing growth behaviour, survival, differentiation and possible malignant transformation of retrovirally modified HSCs in surrogate assays, as well as the influence of the surrounding microenvironment, could be critical. Elucidating the specific molecular and cellular properties that mediate formation and survival of leukaemia initiating cells (LIC) under optimal and sub-optimal conditions is an essential step toward creating efficient approaches for improved therapeutic leukaemia treatment^4,5^.

Constitutive co-expression of TFs such as *Hoxa9* and *Meis1* (H9M) is a conventional approach to transform haematopoietic cells *ex vivo*^6^. *Hoxa9* is critical for the development and self-renewal of healthy HSCs, but its ectopic co-expression with *Meis1* in haematopoietic stem and progenitor cells leads to rapid acute myeloid leukaemia (AML) onset in mice^7,8^. The number and fate impact of potential *Hoxa9* and *Meis1* collaborating genes is not completely understood.

Here, we developed tetracycline-regulated multimodal retroviral vectors (MRVs) based on a gammaretroviral vector (GV) backbone^9^, with the tetracycline-regulated promoter (TRP) T11^10^ inserted into the U3 self-inactivating (SIN) deletion of the 3′ long terminal repeat (LTR). After provirus integration into the host genome, constitutive *Hoxa9* expression should be supplemented by inducible *Meis1* expression in the presence of DOX. MRV design using GV backbone allows drug-controlled co-expression of selected TFs in combination with a potential propensity to activate neighbouring cellular sequences in target cells after MRV integration. It was shown before that SIN GV might transform cells ex vivo by insertional mutagenesis (IM) when strong internal retroviral enhancers/promoters are used^11^.

We established a robust tetracycline-regulated ex vivo model based on conditionally immortalised (ci) myeloid progenitor cell lines co-expressing *Hoxa9* and *Meis1* (H9M-ciMP) in the presence of DOX. The MRV design and DOX-dependent selection approach obtained retroviral insertion (RVI) sites that potentially collaborate with *Meis1*/*Hoxa9* to define H9M-ciMP formation and fate*.* Among them, RVI into the first intron of the adhesion molecule *Ninj2* (nerve injury-induced protein 2)^12^ resulted in multiple gene dysregulations within the locus. The LIC capacities of H9M-ciMPs that had *Ninj2* RVI and were cultured under myelomonocytic^2^ or stem cell maintaining^13^ conditions were evaluated in a murine serial transplantation model, which revealed the rapid development of DOX-dependent AML.

We analysed H9M-ciMP growth behaviour, immunophenotype and morphology under different microenvironmental conditions ex vivo: myelomonocytic^2^, stem cell maintaining^13^ and T-lymphoid based on co-culture with OP9 stromal cells expressing the Notch ligand Delta-like 1 (OP9-DL1)^14,15^. We demonstrated that exponential growth and myeloid differentiation arrest are H9M-ciMP hallmarks in the presence of DOX under tested microenvironmental conditions ex vivo. After DOX removal and further reapplication, myelomonocytic conditions supported the efficient restoration of immature H9M-ciMP exponential growth.

The established tetracycline-regulated H9M-ciMP system can be efficiently employed to reveal new events that collaborate with *Hoxa9* and *Meis1* and define intrinsic and extrinsic signalling pathways that regulate immortalised cell line fate ex vivo and in vivo.

## Results

### Generation of H9M-ciMP cell lines co-expressing *Hoxa9* and *Meis1* in the presence of DOX

**Figure 1:**
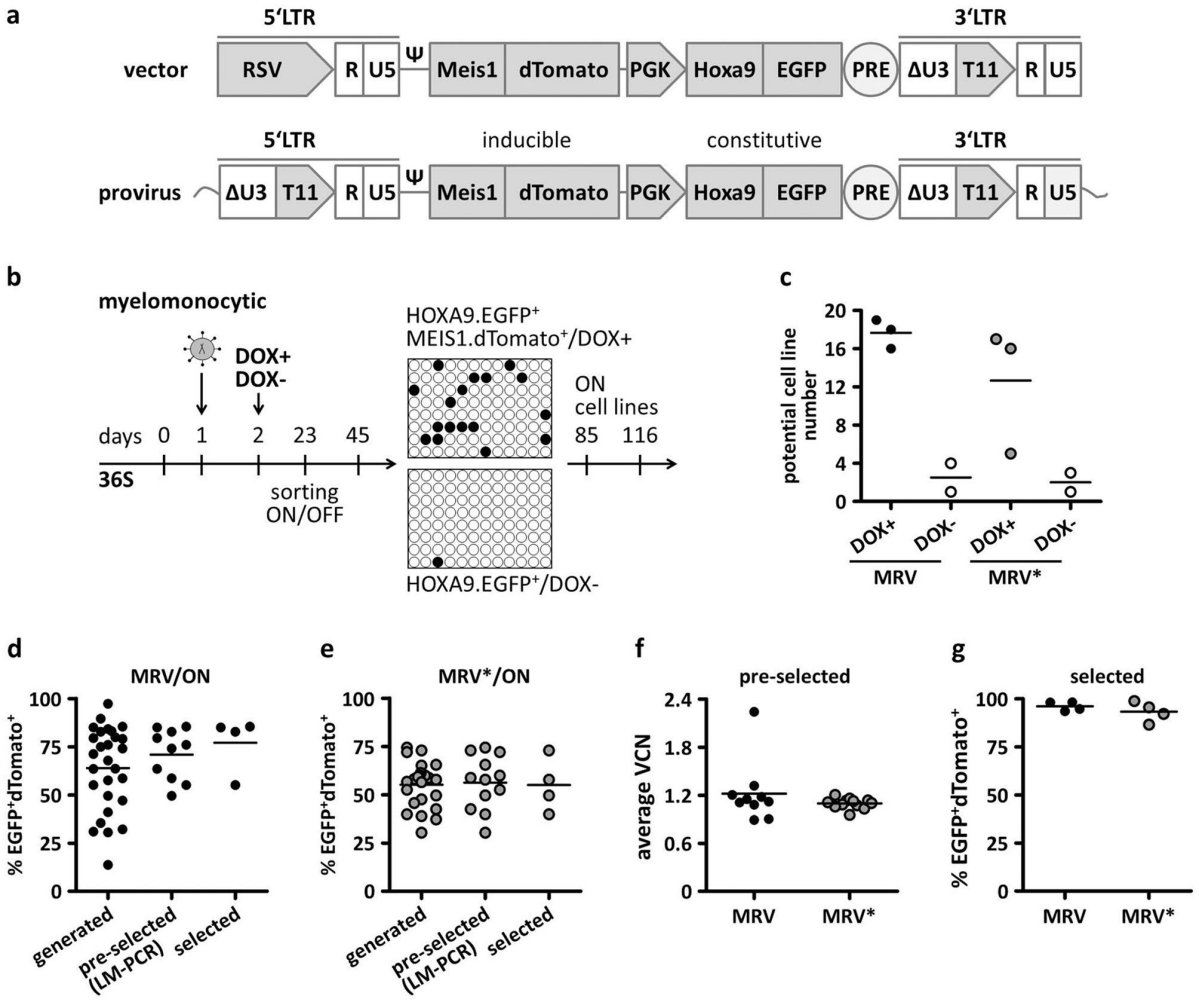
Generation of conditionally immortalised myeloid progenitor cell lines co-expressing *Hoxa9* and *Meis1* in the presence of doxycycline. (**a**) Schematic representation of multimodal retroviral vector and integrated provirus to enable constitutive *Hoxa9*.*EGFP* and inducible *Meis1*.*dTomato* gene co-expression. RSV, Rous sarcoma virus promoter; Δ, SIN configuration with partially deleted U3 of the 3′ long terminal repeat (LTR); ψ, packaging signal; PRE, post-transcriptional regulatory element; EGFP, dTomato, fluorescent proteins. (**b**) Experimental design. Murine Rosa26rtTA lineage negative cells were transduced on day 1 under myelomonocytic conditions (36S) without doxycycline (DOX). On the next day, cells were split into DOX untreated (DOX-, OFF) and DOX treated cultures, which were supplemented with 1 µg/mL DOX (DOX+ , ON). HOXA9.EGFP^+^/MEIS1.dTomato^+^ double-positive cells (DOX+) and HOXA9.EGFP^+^ (DOX-) were sorted on days 13–23 after transduction followed by a limiting dilution assay to select stable H9M-ciMPs. (**c**) The number of potential cell lines for DOX+ and DOX- conditions (~ day 45 after transduction). The experiment was performed independently for multimodal retroviral vectors with (MRV) or without (MRV*) LM-PCR linker in three biological replicates for DOX+ and two biological replicates for DOX- conditions (one biological replicate / one 96-well plate). (**d-e**) The number of generated, pre-selected for LM-PCR analysis and selected cell lines for MRV and MRV* vectors under DOX supplementation. (**f**) Average vector copy number (VCN) determined for pre-selected (*n* = 10 for MRV, *n* = 12 for MRV*) H9M-ciMPs. (**g**) Gene expression levels of EGFP^+^dTomato^+^ double-positive cells in selected (*n* = 8) H9M-ciMPs after cryopreservation (pre-cultured ~ 3 months in 36S conditions before cryopreservation). Horizontal lines represent the mean values. Each data point represents the result of an individual cell line. LM-PCR, ligation-mediated PCR. H9M-ciMPs, conditionally immortalised myeloid progenitor cell lines engineered to co-express *Hoxa9* and *Meis1*. (**d**–**g**) generated in Prism 5 (GraphPad software, San Diego, CA).

To establish H9M-ciMP cell lines as a robust system to study conditional HSC immortalisation and differentiation, we developed tetracycline-regulated MRVs based on a GV vector backbone^9^, containing constitutive and inducible gene expression cassettes. To track the constitutive *Hoxa9* and inducible *Meis1* expression, we used Enhanced Green Fluorescent Protein (*EGFP*) and red fluorescent protein dTomato, respectively. The *Hoxa9.EGFP* constitutive cassette was cloned under the control of the murine phosphoglycerate kinase (mPGK) promoter^1^. The TRP T11^10^ was inserted into the U3 SIN deletion of the 3′ LTR of the same vector. Thus, constitutive *Hoxa9.EGFP* expression should be supplemented by inducible *Meis1.dTomato* expression in the presence of DOX (Fig. 1a). The MRV was equipped with a 304 bp linker cloned in front of the T11 TRP to provide an additional sequence for ligation-mediated PCR (LM-PCR) primer design to investigate RVIs. Additionally, an MRV vector without a linker (MRV*) was used.

Murine haematopoietic progenitor cells (HPC) from Rosa26rtTA-nls-Neo2 (Rosa26rtTA) mice^16–18^ were transduced with MRV or MRV* (in three biological replicates) under myelomonocytic cytokine conditions (36S: IL3, IL6, SCF) without DOX. One day after transduction, cultures were split and half of the cells were treated with 1 µg/mL DOX (DOX+)^1,2^ (Fig. 1b). The other half was cultured further without DOX (DOX-) as a negative control (Fig. 1b). This experimental design ensures similar transduction levels for different DOX conditions for cells that are derived from the same biological replicate and enables identification of the insertional mutagenesis targets that potentially collaborate with *Hoxa9* and *Meis1* under DOX supplementation. Thus, double-positive HOXA9.EGFP^+^/MEIS1.dTomato^+^ (DOX+) or single-positive HOXA9.EGFP^+^/MEIS1.dTomato^−^ (DOX-) cells were sorted, and limiting dilution assays were accomplished, which resulted in a higher number of potential cell lines for DOX+ conditions (Fig. 1b,c). Under DOX- conditions, cells did not proliferate sufficiently.

Cell lines generated in the presence of DOX were cultured under myelomonocytic 36S (DOX+) conditions and the cell lines that showed constant double-positive HOXA9.EGFP^+^/MEIS1.dTomato^+^ expression over time and stable proliferation were pre-selected (Fig. 1d,e; Supplementary Table S1). Of note, at this pre-selection step, the normally applied DOX concentration (1 µg/mL), which is sensitive to cell density, may not have provided a stably high level of double-positive HOXA9.EGFP^+^/MEIS1.dTomato^+^ expression in all cultures due to varying H9M-ciMP cell number and proliferation rate (Fig. 1d,e).

Average vector copy numbers (VCN) for all 36S pre-selected H9M-ciMPs were determined as one (1.1 ± 0.1, mean ± SD, *n* = 21) except for cell line #12 (Fig. 1f). The insertional analysis of 22 pre-selected H9M-ciMP cell lines demonstrated the relevant pattern and number of specific RVIs (Supplementary Fig. S1; Supplementary Table S1, S2). As it was previously shown that multiple distal genes surrounding a single insertion event could be dysregulated^19–22^, we annotated genes located within ~  ± 250 Kb from the RVI (Supplementary Table S2). Some H9M-ciMP cell lines obtained from the same biological replicates demonstrated identical RVIs (e.g. #1 and #5) into the first intron of *Ninj2*^12^ (Supplementary Fig. S1; Supplementary Table S1, S2). Other H9M-ciMP of different origin exhibited RVIs into the same gene locus at a different position, e.g. *Cmah* (cytidine monophospho-N-acetylneuraminic acid hydroxylase)^23,24^ (Supplementary Table S2). Remarkably, the *Cmah* locus also revealed identical RVIs in related H9M-ciMPs #24, 25, 28 and 29, which originated from the same biological replicate (Supplementary Table S1, S2). Interestingly, neighbouring genes surrounding the RVIs in all H9M-ciMPs were found to be involved in oncogenesis, immune response, developmental and/or differentiation processes, or were evolutionary essential metabolic genes (Supplementary Table S2).

To study H9M-ciMPs under myelomonocytic DOX+/DOX- conditions, we focused on 8 H9M-ciMP cell lines originating from pre-selected MRV and MRV* derived cell lines. These cell lines had RVIs in the vicinity of the *Ninj2* gene promoting neurite outgrowth^12^, *Vps45 (*vacuolar protein sorting 45)/*Otud7b* (OTU domain containing 7B)^25,26^, *Plet1* (placenta expressed transcript 1)^27^, *Thada* (thyroid adenoma associated)^28^, *Cmah*^23,24^, and *Chsy1* (chondroitin sulfate synthase 1)^29^ (Supplementary Table S1, S2).

After cryopreservation, these eight H9M-ciMP cell lines demonstrated a high percentage of EGFP^+^dTomato^+^ double-positive cells (94.7% ± 4.1%, mean ± SD, *n* = 8) (Fig. 1g) and were selected for further characterisation using DOX ON/OFF/re-ON conditions.

### Myelomonocytic conditions supported the reversibility of DOX-dependent H9M-ciMP differentiation

**Figure 2:**
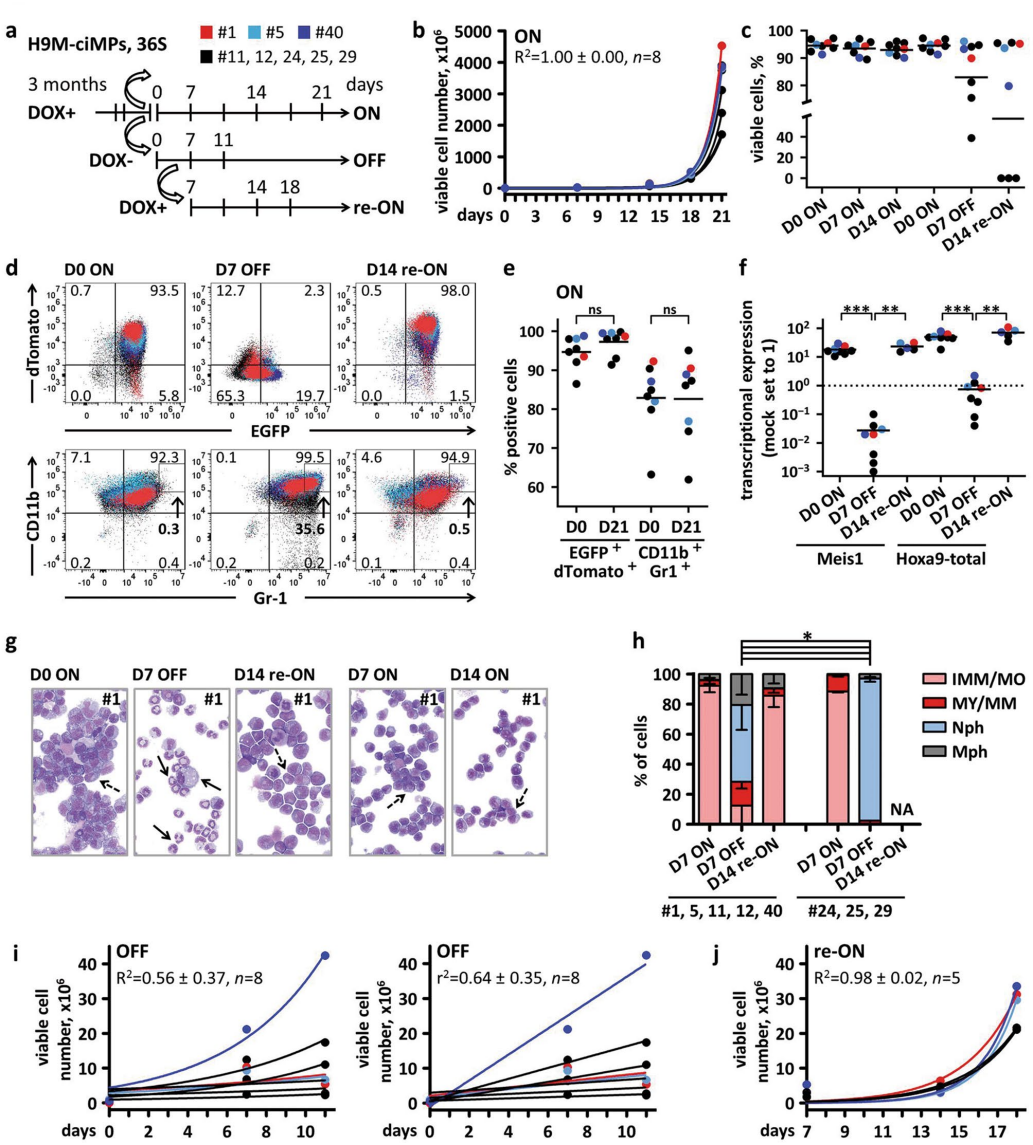
Characterisation of H9M-ciMPs under myelomonocytic conditions (36S) and different doxycycline application. (**a**) Schematic presentation of 1 µg/mL doxycycline application (DOX+ , ON), removal (DOX-, OFF) and reapplication (DOX+ , re-ON). 5 × 10^5^ DOX+ cells were taken for parallel ON and OFF experiments (initial day (D) 0); 1/4 DOX- cells were taken for parallel re-ON experiment (initial D7). Cell numbers for growth curves were calculated based on initial days. Indicated days correspond to time points taken for analysis. H9M-ciMPs #1,5,40 are marked in red, light blue and blue, respectively; #11,12,24,25,29 are marked in black. The H9M-ciMP colour coding applies to all parts of the figure. (**b**) Exponential growth equation under ON conditions. R^2^, R-squared value. (**c**) Percentages of viable cell numbers under different DOX conditions, *n* = 8. (**d**) Dot plot overlays to present expression of EGFP^+^dTomato^+^ and CD11b^+^Gr-1^+^ (frequency percentages are given for H9M-ciMPs #1). Arrows indicate percentages of CD11b^high^Gr-1^high^ cells. (**e**) Percentages of EGFP^+^dTomato^+^ and CD11b^+^Gr-1^+^ cells for D0/ON and D21/ON, *n* = 8. (**f**) Relative transcriptional expression of *Meis1* and *Hoxa9-*total in H9M-ciMPs upon DOX ON/OFF/re-ON conditions. Expression levels in non-transduced non-cultured lineage negative cells (Mock) were set to 1. The mean values are represented by horizontal lines, *n* = 8. (**g**) Cytospin analysis (May-Grünwald/Giemsa staining, magnification × 80) of H9M-ciMP #1 under different DOX supplementation. Dashed black arrows indicate immature myeloid cells; solid black arrows indicate neutrophils or macrophages. (**h**) Histogram presentation of differential cell counts for H9M-ciMPs under different DOX conditions. #1,5,11,12,40, *n* = 5; #24,25,29, *n* = 3. IMM/MO, immature cells: myeloblast-, promyelocyte-, monoblast-, promonocyte- and monocyte-like cells; MY/MM, myelocyte-, metamyelocyte-like; Nph, band neutrophil, segmented neutrophil; Mph, macrophage. Data are represented as mean ± SD. (**i**) Exponential growth equations and linear regression after DOX removal. (**j**) Exponential growth equation after DOX reapplication. The values R^2^ and r^2^ are indicated as a measure of goodness-of-fit of the exponential and linear growth models. Each data point represents the result of an individual cell line. ns, not significant, *P*>0.05; *, *P* < 0.05; **, *P* < 0.01; ***, *P* < 0.001 (non-parametric two-tailed Mann–Whitney test). H9M-ciMPs, conditionally immortalised myeloid progenitors co-expressing *Hoxa9*/*Meis1*. Figure 2c,e,f, generated in Matplotlib 3.1.1 https://matplotlib.org; (**d**) generated in PowerPoint using plots from Flowjo 10 (Tree Star, Ashland, OR); (**g**) generated in PowerPoint using pictures from NDP.view 2.8.24 viewing software U12388-01, https://www.hamamatsu.com/eu/en/product/type/U12388-01/index.html.

We investigated the H9M-ciMP growth behaviour and differentiation status upon ON/OFF/re-ON switching under myelomonocytic conditions (Fig. 2, Supplementary Fig. S2). DOX+ conditions (ON) resulted in very rapid exponential growth, with increased viable cell numbers of up to 6767.8 ± 1848.9 fold (mean ± SD, *n* = 8) within 21 days (D) (Fig. 2a–c). At D0/ON, co-expression of HOXA9.EGFP and MEIS1.dTomato (94.7% ± 4.1%, mean ± SD, *n* = 8) was accompanied by high *Meis1* and *Hoxa9* transcriptional co-expression (Fig. 2d–f; Supplementary Fig. S2a,b).

After DOX withdrawal (OFF) at D7, the percentage of HOXA9.EGFP^+^/MEIS1.dTomato^+^ double-positive cells decreased to 1.5% ± 1.0% (mean ± SD, *n* = 8) with no significant change of VCNs (Fig. 2d; Supplementary Fig. S2b,c). However, the percentage of constitutively expressed HOXA9.EGFP^+^ cells also decreased (28.6% ± 32.6%, mean ± SD, *n* = 8), although to a different degree in distinct H9M-ciMPs (Fig. 2d; Supplementary Fig. S2d). In line with this, not only *Meis1* but also *Hoxa9* transcriptional expression decreased when compared to mock non-transduced non-cultured Lin^−^ cells (Fig. 2f). It was previously described for similar vector systems that DOX induction leads to increased gene expression by both tetracycline-regulated T11 and constitutive mPGK promoters located in the same vector, and DOX removal results in downregulation of expression under both promoters^1^.

This could be explained by the presence of “read-through” transcript driven by the T11 promoter, spanning the *Hoxa9* region and supplementing constitutive mPGK *Hoxa9* transcriptional expression under DOX application (Supplementary Fig. S3a). Thus, here we measured the relative transcriptional expression of *Hoxa9* is cumulative and determined as “Hoxa9-total” (Fig. 2f; Supplementary Fig. S3a). After drug-removal (D7/OFF), “read-through” transcription expression is efficiently down-regulated (Supplementary Fig. S3), and only constitutive *Hoxa9* transcript is abundant, explaining the drop in *Hoxa9* transcriptional expression (Fig. 2f).

To track H9M-ciMP differentiation, we used CD11b and Gr-1 marker expression levels to directly correlate differentiation and maturation of cells of the myelomonocytic lineage^30,31^ and morphology analysis^32^.

In the presence of DOX, the percentages of CD11b^+^Gr-1^+^ cells were stable and did not reveal significant differences between the initial (D0/ON) and end (D21/ON) time points of exponential growth (Fig. 2e). At D0/ON, the percentage CD11b^high^Gr-1^high^ cells was only 0.4% ± 0.3% (mean ± SD, *n* = 8) (Fig. 2d; Supplementary Fig. S2e). In the presence of DOX, H9M-ciMPs showed blockade of myelomonocytic differentiation characterised by the high percentage of immature myelomonocytic cells (90.7% ± 3.8%, mean ± SD, *n* = 8, D7/ON) (Fig. 2g,h).

After DOX withdrawal and *Meis1*/*Hoxa9* transcriptional downregulation (D7/OFF), morphology analysis revealed myeloid differentiation into mostly neutrophils associated with the increased population of CD11b^high^Gr-1^high^ cells (37.5% ± 20.1%, mean ± SD, *n* = 8) (Fig. 2g,h; Supplementary Fig. S2e).

After seven days of DOX reapplication (re-ON) (D14 from the initial day of the experiment, see D14 re-ON at Fig. 2a), only five (#1, 5, 11, 12, 40) from eight H9M-ciMPs survived DOX removal, as tracked by viable cell number (Fig. 2c). In these “reversible” cell lines, the transcriptional *Hoxa9* and *Meis1* co-expression level was re-established as well as the percentage of HOXA9.EGFP^+^/MEIS1.dTomato^+^ double-positive cells (97.4% ± 1.4%, mean ± SD, *n* = 5) (Fig. 2a,d,f; Supplementary Fig. S2b). The myeloid progenitor morphology returned and percentage of CD11b^high^Gr-1^high^ cells decreased to only 1.4% ± 0.9% (mean ± SD, *n* = 5) (Fig. 2d,g,h; Supplementary Fig. S2e).

Interestingly, “reversible” H9M-ciMP cell lines demonstrated significantly higher percentages of HOXA9.EGFP^+^ cells at D7/OFF when compared to “nonreversible” cell lines (#24, 25, 29) (Supplementary Fig. S2d). Accordingly, at D7/OFF, “reversible” cell lines demonstrated significantly higher percentages of immature myelomonocytic cells, while the majority of “nonreversible” cells differentiated towards the band and/or segmented neutrophils or macrophages (Fig. 2h). Even at D11/OFF, “reversible” H9M-ciMP revealed a mixture of immature and differentiated myelomonocytic cells (Supplementary Fig. S4), thus explaining the lack of exponential or linear expansion of H9M-ciMPs under DOX- conditions (Fig. 2i). Prolonged survival of HOXA9.EGFP^+^ immature myelomonocytic cells under suboptimal DOX- conditions in “reversible” cell lines could be essential for the rapid reconstitution of an exponentially growing pool of immature myeloid cells after DOX reapplication (Fig. 2j).

Thus, myelomonocytic conditions supported the efficient and rapid reconstitution of exponentially proliferating immature myeloid cells after DOX removal and reapplication, but not for all investigated H9M-ciMP cell lines. Such survival heterogeneity could be determined by intrinsic properties of “nonreversible” H9M-ciMPs (#24, 25, 29) obtained from the same biological replicate, characterised by identical RVIs into the evolutionary remarkable *Cmah* gene^23,24^ and showing rapid terminal differentiation after DOX removal (Supplementary Table S1, S2, Fig. 2h). Murine models indicate the impact of *Cmah* on the innate immune response suggesting that loss of *CMAH* during human evolution primed the monocyte-macrophage lineage toward inflammatory and phagocytic state^33^. It was also shown that inactivation of murine *Cmah* was associated with enhanced atherosclerosis or impaired hearing^23,24^.

### Distinct H9M-ciMPs demonstrated exponential growth under stem cell maintaining cytokine conditions in the presence of DOX

**Figure 3:**
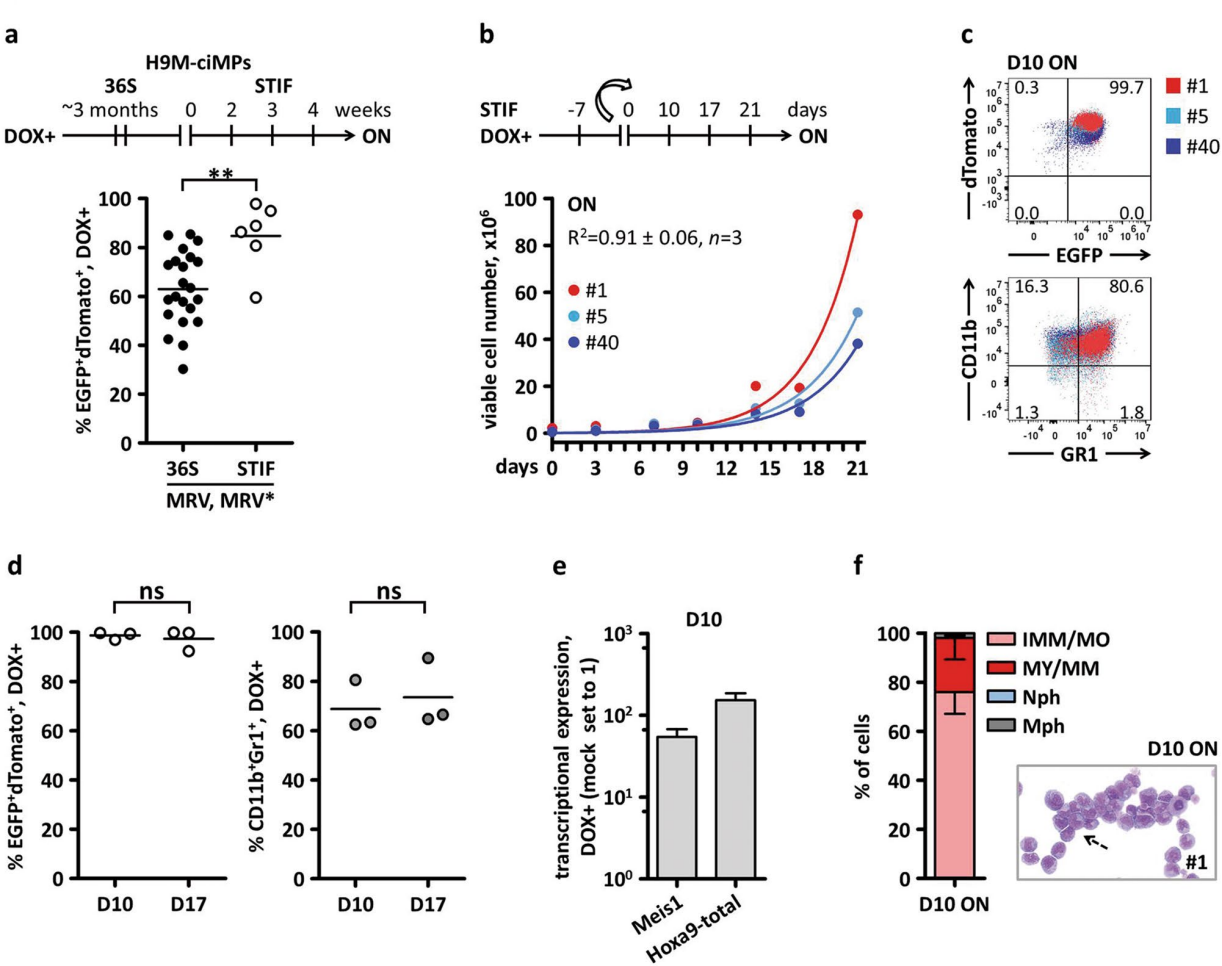
Selection and characterisation of H9M-ciMPs under stem cell maintaining conditions and doxycycline application. (**a**) Schematic presentation of H9M-ciMP selection under stem cell maintaining conditions (STIF) and 1 µg/mL doxycycline (ON) supplementation. H9M-ciMPs resulting from MRV or MRV* experiments were cultured under STIF conditions. MRV, MRV*, multimodal retroviral vectors with or without LM-PCR linker, respectively. Each data point represents the result of an individual cell line. Horizontal lines represent the mean values. (**b**) Exponential growth equation for H9M-ciMPs cultured under STIF/ON conditions. R^2^, the R-squared value. (**c**) Dot plot overlays to present expression of CD11b^+^Gr-1^+^ in H9M-ciMPs at day 10 of culture under STIF/ON conditions (frequency percentages are given for H9M-ciMP cell line #1). (**d**) Percentages of EGFP^+^dTomato^+^ and CD11b^+^Gr-1^+^ cells for D10/ON and D17/ON STIF conditions, *n* = 3. Each data point represents the result of an individual cell line. Horizontal lines represent the mean values. ns, not significant, *p* > 0.05 (non-parametric two-tailed Mann–Whitney test). (**e**) Histogram presentation of relative transcriptional expression of *Meis1* and *Hoxa9* (corresponds to *Hoxa9*-total) in H9M-ciMPs. Expression levels in non-transduced non-cultured lineage negative cells (Mock) were set to 1. Data are represented as mean ± SD, *n* = 3. (**f**) Histogram presentation of differential cell count for H9M-ciMPs under STIF/ON conditions. IMM/MO, immature cells: myeloblast-, promyelocyte-, monoblast-, promonocyte- and monocyte-like cells; MY/MM, myelocyte-, metamyelocyte-like; Nph, band neutrophil, segmented neutrophil; Mph, macrophage. Data are represented as mean ± SD, *n* = 3. H9M-ciMPs, conditionally immortalised myeloid progenitor cell lines engineered to co-express *Hoxa9* and *Meis1.* D, day. For (**b**) and (**c**) H9M-ciMPs #1, 5 and 40 are marked in red, light blue and blue, respectively. (**a**, **d**) generated in Prism 5 (GraphPad software, San Diego, CA); (**c**) generated in PowerPoint using plots from Flowjo 10 (Tree Star, Ashland, OR).

It was previously shown that HSCs and LICs survival and proliferation ex vivo could be defined by similar cytokine conditions. For instance, the stem cell maintaining cytokine combination significantly promoted both HSC and LIC proliferation^13,34,35^. To address this, we investigated H9M-ciMP fate and growth behaviour in the presence of DOX under stem cell maintaining^13,34^ conditions (STIF: SCF, TPO, IGF2, FGF1). Only 6 of 22 investigated H9M-ciMP cell lines demonstrated sufficient proliferation (Fig. 3a; Supplementary Table S1). Selective survival could be explained by the requirement of additional insertional events^34^, e.g. identical RVI into the 1st intron of adhesion molecule *Ninj2* in H9M-ciMP #1 and #5, suggesting that these cell lines may have arisen from a common ancestor (Supplementary Fig. S1a; Supplementary Table S2). *Ninj2* was previously reported to contribute to neurite outgrowth and enhance growth, survival and proliferation of glioma and colorectal cancer cells^12,36,37^. Another candidate that survived, cell line #40, was characterised by RVI into the vicinity of the *Chsy1* gene, which is involved in neutrophil and macrophage survival and differentiation^29^.

Cell lines #1, #5 and #40 are “reversible” H9M-ciMPs as shown in ON/OFF/re-ON experiments under 36S conditions (Fig. 2c,f,j). They demonstrated exponential growth in the second independent experiment under STIF conditions, however, the proliferation rate was relatively low (Fig. 3b). Thus, the first point available for analysis was D10 after initiation of the experiment. Percentages of EGFP^+^dTomato^+^ and CD11b^+^Gr-1^+^ cells at D10 (98.7% ± 1.5%, mean ± SD, *n* = 3; 69.9% ± 9.5%, *n* = 3 respectively) did not reveal significant differences from D17 (Fig. 3c,d). The real-time quantitative PCR (RT-qPCR) confirmed the relative transcriptional upregulation of *Hoxa9* and *Meis1* in H9M-ciMPs under STIF cytokine conditions and DOX (1 μg/mL) supplementation when compared to mock non-transduced controls (Fig. 3e).

Interestingly, at D10/ON under STIF conditions, H9M-ciMPs demonstrated only 76.0% ± 8.9% (mean ± SD, *n* = 3) of immature myelomonocytic cells and up to 22.0% ± 8.7% of more differentiated myelocytes and metamyelocytes^32^ (Fig. 3f). This may indicate that an adaptative phase could take place, influencing the exponential growth rate (Fig. 3b). More long term experiments are required to fully characterise the H9M-ciMP immunophenotype and cell morphology under STIF conditions.

### Murine serial transplantation of H9M-ciMPs bearing RVI into the *Ninj2* locus cultured under myelomonocytic or STIF cytokine conditions resulted in DOX-dependent AML development

To investigate if the H9M-ciMPs cultured under myelomonocytic or stem cell maintaining conditions are transplantable in vivo, we performed serial murine transplantation. To select cell lines, we considered the following: H9M-ciMP “reversibility” after DOX reapplication under myelomonocytic conditions, and increased survival under STIF conditions.

**Figure 4:**
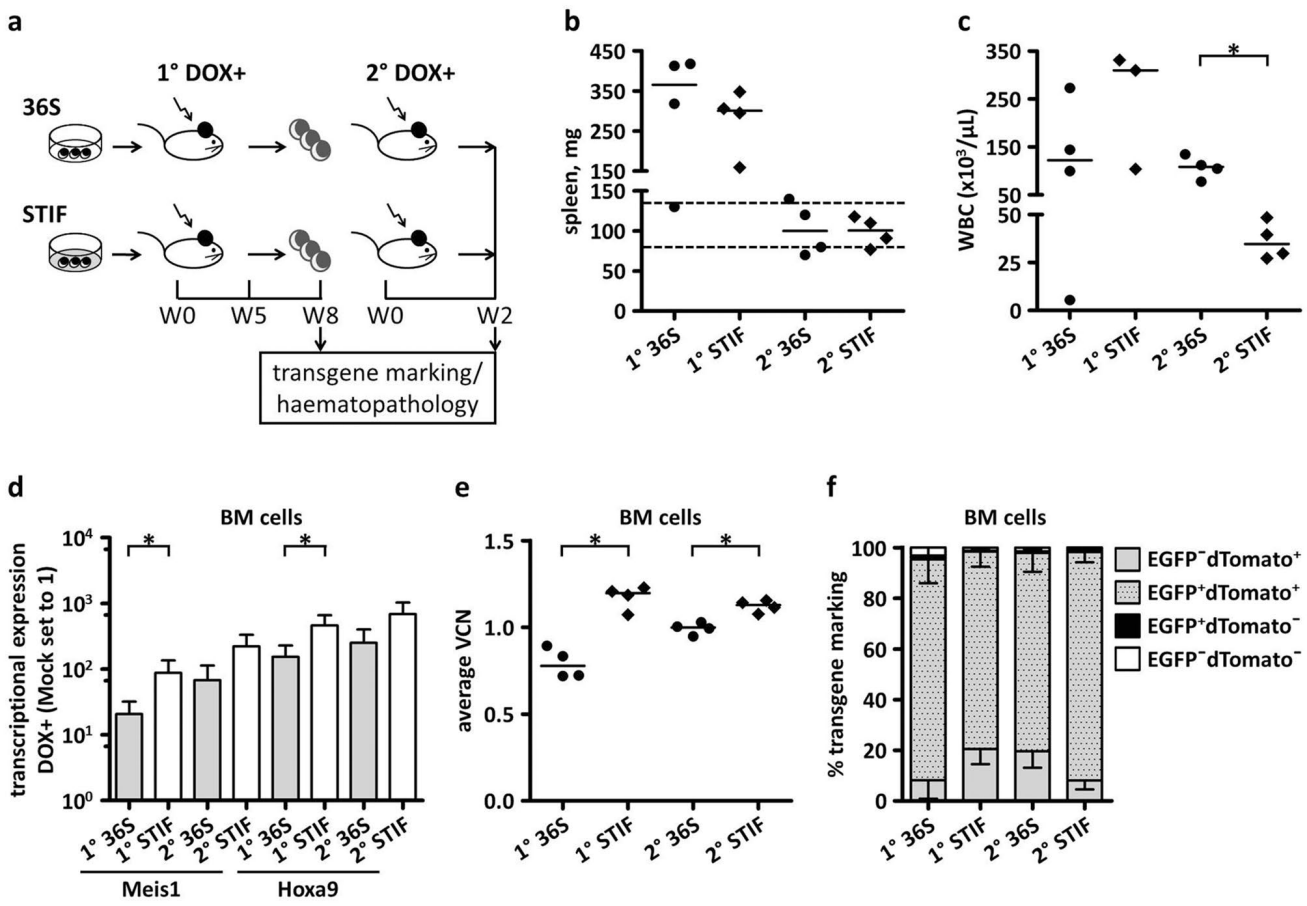
Drug-controlled murine serial transplantation of H9M-ciMPs. (**a**) Experimental design: H9M-ciMPs cultured in myelomonocytic (36S) or stem cell maintaining (STIF) cytokine conditions were transplanted into primary (1°) recipient mice and symptomatic mice were analysed 8 weeks after transplantation. Bone marrow (BM) cells from selected 1° 36S and STIF recipients were transplanted into secondary (2°) recipients and analysed after 2 weeks. 1° and 2° recipients received doxycycline (DOX). (**b**) Spleen weight of 1° and 2° 36S and STIF recipients. Dashed lines indicate the normal spleen weight range. (**c**) Analysis of white blood cell (WBC) counts in 1° and 2° recipients (WBC data for 1° STIF recipient #227 are not presented due to a technical problem). (**d**) Histogram presentation of *Meis1* and *Hoxa9* transcriptional expression in BM cells of 1° and 2° 36S and STIF recipients. The expression level of the control non-transplanted DOX-treated murine cells (Mock) was set to 1. Data are represented as mean ± SD for the animals in each group, *n* = 4. *, *P* < 0.05 (non-parametric two-tailed Mann–Whitney test). (**e**) Average vector copy number (VCN) in total BM cells of individual 1° and 2° 36S and STIF recipients. (**f**) Histogram presentation of percentages of EGFP and/or dTomato expression in BM cells of 1° and 2° 36S and STIF recipients. Data are represented as mean ± SD for the animals in each group, *n* = 4. Percentages of EGFP^+^dTomato^+^ cells were compared (1° 36S *versus* 1° STIF, 2° 36S *versus* 2° STIF). The differences are not statistically significant, *P* > 0.05. For (**b**) and (**c**), horizontal lines indicate median values; for (**e**), horizontal lines indicate mean values. Each data point represents the result of an individual animal, *n* = 4, *, *P* < 0.05. Comparisons were made using the non-parametric two-tailed Mann–Whitney test. H9M-ciMPs, conditionally immortalised myeloid progenitor cell lines engineered to co-express *Hoxa9* and *Meis1*; W, week. (**b**, **c**, **e**) generated in Prism 5 (GraphPad software, San Diego, CA).

We chose cell lines #1 and #5, as they fulfil these criteria and have identical insertional patterns (Supplementary Fig. S1a, Supplementary Table S1, S2). Cell lines #1 and #5 were pooled together after culturing under 36S or STIF conditions (Material and Methods) and used as donor cells (Fig. 4a, Supplementary Table S1). DOX was administered to all primary (1°) and secondary (2°) recipients before and throughout the experiment.

Irrespective of cytokine conditions, all 1° recipients at week eight following transplantation developed AMLs characterised by remarkably increased spleen weights (36S: 365.5 ± 134.6 mg; STIF: 300.5 ± 81.9; median ± SD, *n* = 4) and peripheral white blood cell (WBC) numbers (36S: 122.1 ± 111.3 × 10^3^/µL, *n* = 4; STIF: 310.0 ± 125.8 × 10^3^/µL, *n* = 3; median ± SD) (Fig. 4b,c).

2° recipients transplanted with bone marrow (BM) from selected 1° 36S (#222) or STIF (#225) animals (Supplementary Table S1) in the presence of DOX developed very rapid (within two weeks) AMLs characterised by normal spleen weight, but high numbers of peripheral WBCs (36S: 108.3 ± 23.4 × 10^3^/µL, STIF: 34.6 ± 9.7 × 10^3^/µL; median ± SD, *n* = 4) (Fig. 4b,c).

Transcriptional expression demonstrated significantly higher levels of *Meis1* and *Hoxa9* in BM cells of DOX-treated 1° STIF animals when compared to 1° 36S mice (Fig. 4d). In line with this observation, average VCNs in total BM were close to one, but significantly higher in BM of 1° and 2° STIF mice in comparison with 36S animals: (1° STIF 1.2 ± 0.1 versus 1° 36S 0.8 ± 0.1; 2° STIF 1.1 ± 0.03 versus 2° 36S 1.0 ± 0.03; mean ± SD, *n* = 4) (Fig. 4e). However, no statistically significant differences in the percentages of EGFP^+^dTomato^+^ cells were observed in BM from 36S and STIF 1°and 2° mice (Fig. 4f; Supplementary Fig. S5).

**Figure 5:**
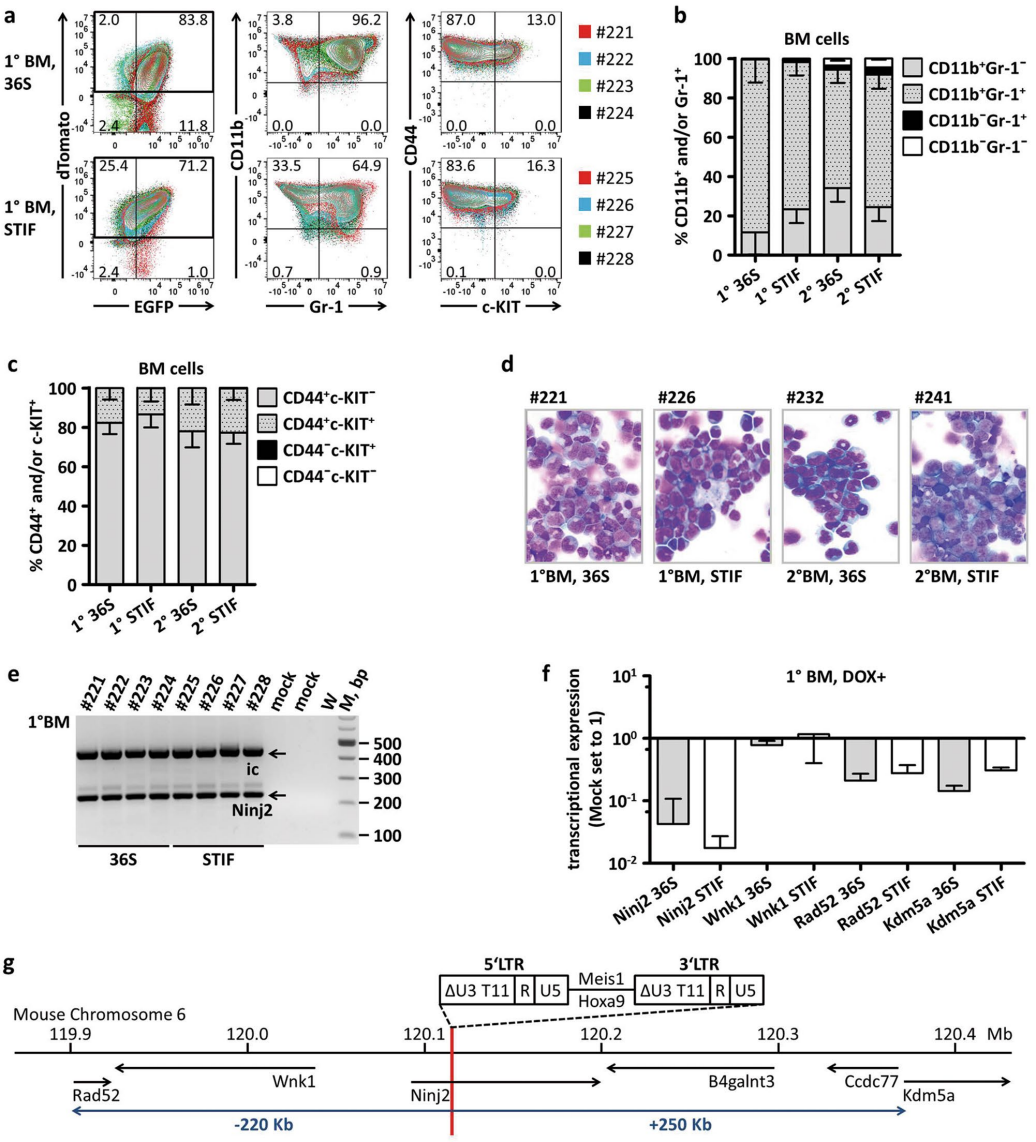
Immunophenotype, morphology and insertional analysis of doxycycline-dependent H9M-ciMP induced AMLs. (**a**) Strategy of bone marrow (BM) immunophenotype analysis of doxycycline-treated primary (1°) recipients transplanted with H9M-ciMPs cultured under myelomonocytic (36S) or stem cell maintaining (STIF) conditions. Flow cytometry plot overlays to present expression of CD11b/Gr-1 and CD44/c-KIT in the EGFP^+^dTomato^+^ and EGFP^−^dTomato^+^ cells. Frequency percentages are given for recipients #224 (36S) and #228 (STIF). (**b–c**) Histogram presentation of percentages of CD11b^+^ and/or Gr-1^+^ (**b**) and CD44^+^ and/or c-KIT^+^ (**c**) positive cells in gated EGFP^+^dTomato^+^ and EGFP^−^dTomato^+^ cells from BM of 1° and secondary (2°) 36S and STIF recipients. Data are represented as mean ± SD for animals in each group, *n* = 4. (**d**) Cytospin analysis (May-Grünwald/Giemsa staining, magnification × 60) of BM cells from selected 1° and 2° 36S and STIF recipients. (**e**) Ligation-mediated PCR analysis demonstrating insertion sites in BM cells of 1° 36S and STIF recipients. W, water; M, 100 bp marker; bp, base pairs; ic, internal control; mock, BM cells from non-transplanted doxycycline-treated mice. (**f**) Histogram presentation of transcriptional expression for genes located in the *Ninj2* locus in BM cells of 1° 36S and STIF recipients. The expression level of the control non-transplanted DOX-treated murine cells (Mock) was set to 1. Data are represented as mean ± SD, *n* = 4 (for *Ninj2*), *n* = 3 (for other genes). (**g**) Genes located within ± 250 kb from retroviral insertion in the *Ninj2* locus. Scale based on the Ensembl database, release 98—August 2020. H9M-ciMPs, conditionally immortalised myeloid progenitor cell lines engineered to co-express *Hoxa9* and *Meis1*; AML, acute myeloid leukaemia.

EGFP^+^dTomato^+^ and EGFP^−^dTomato^+^ cells infiltrating BM of 1° and 2° 36S and STIF mice were highly positive for the myeloid marker CD11b (1° 36S: 99.5% ± 0.5%, mean ± SD, *n* = 4; 1° STIF: 98.2% ± 0.9%, *n* = 4) (Fig. 5a,b; Supplementary Fig. S5b). Percentages of cells positive for the myeloid marker Gr-1 were lower: 88.1% ± 11.5% (mean ± SD, *n* = 4, 1° 36S) to 76.2% ± 7.3% (*n* = 4, 1° STIF) and thus consistent with previously described murine *Hoxa9*/*Meis1* induced AML^7^ (Fig. 5a,b; Supplementary Fig. S5b).

EGFP^+^dTomato^+^ and EGFP^−^dTomato^+^ cells of the spleen, lymph nodes and thymus of 1° 36S and STIF recipients revealed comparable co-expression of CD11b and Gr-1 markers and were mostly negative for B220 and CD25 (Supplementary Fig. S6, S7).

EGFP^+^dTomato^+^ and EGFP^−^dTomato^+^ cells in all haematopoietic organs highly expressed the adhesion molecule CD44 (e.g. BM 1° 36S: 99.9% ± 0.1%, mean ± SD, *n* = 4; BM 1° STIF: 99.9% ± 0.1%, *n* = 4), which was reported to be required for homing and engraftment of murine LICs and related with poor prognosis^38,39^ (Fig. 5a,c; Supplementary Fig. S5b). CD44 was co-expressed with c-KIT, another marker associated with LIC biology^40^, in both 1° and 2° BM cells: 1° 36S: 17.6% ± 5.8%, mean ± SD, *n* = 4; 1° STIF: 13.3% ± 6.7%, *n* = 4 (Fig. 5a,c; Supplementary Fig. S5b).

Morphologically, the BM of 1° and 2° 36S and STIF recipients demonstrated dense infiltration with cells showing myelomonocytic and monoblastic differentiation (Fig. 5d), resembling AML M4/M5, which is commonly associated with MLL translocations and overexpression of *HOXA9* and *MEIS1* in patients^41,42^.

LM-PCR analysis of bone marrow of 1° and 2° 36S and STIF recipients confirmed a single integration into the 1st intron of adhesion molecule *Ninj2* (Supplementary Table S2). The location of this integration was identical to that initially described for H9M-ciMPs #1 and #5 in vitro (Fig. 5e; Supplementary Fig. S1a, S8, S9; Supplementary Table S2), indicating successful engraftment of H9M-ciMP #1 and #5 cultured under 36S or STIF conditions, which resulted in AML.

It was described that genes located distantly from common proviral insertional sites are as frequently deregulated as proximal genes, with multiple genes affected per integration^19–22^. Transcriptional expression analysis of several genes located in the *Ninj2* locus within ~  ± 250 Kb from the RVI demonstrated down-regulation of *Ninj2*, *Rad52* (RAD52 homolog, DNA repair protein)^43^ and epigenetic regulator *Kdm5a* (lysine (K)-specific demethylase 5A)^44^ when compared to non-transplanted DOX-treated murine cells (Fig. 5f,g). *Wnk1* (WNK lysine deficient protein kinase 1)^45^ transcription remained mostly unchanged, suggesting that the degree of downregulation correlated not only with the distance from RVI, but also with forward provirus orientation in regards to gene transcription direction (Fig. 5f,g; Supplementary Table S2).

According to the BloodSpot database^46^, *NINJ2* is also downregulated in human AMLs. BloodSpot is a database that provides gene expression in AML in comparison with corresponding healthy cells and is an aggregated and integrated dataset grouping the results of multiple studies that focus on AML^46^. To conclude, serial transplantations demonstrated that H9M-ciMPs bearing RVI into the *Ninj2* locus possess LIC ability under DOX supplementation and could be defined as ciLICs.

### H9M-ciMPs demonstrated exponential growth of immature myelomonocytic cells under T-lymphoid cytokine conditions in the presence of DOX

A novel tumour suppressor role for Notch signalling in AML was reported recently, suggesting that Notch pathway activation could represent a therapeutic strategy in AML treatment. Thus, co-culture with OP9-DL4 stromal cells resulted in cell cycle arrest, differentiation and apoptosis of AML-initiating cells^47^.

**Figure 6:**
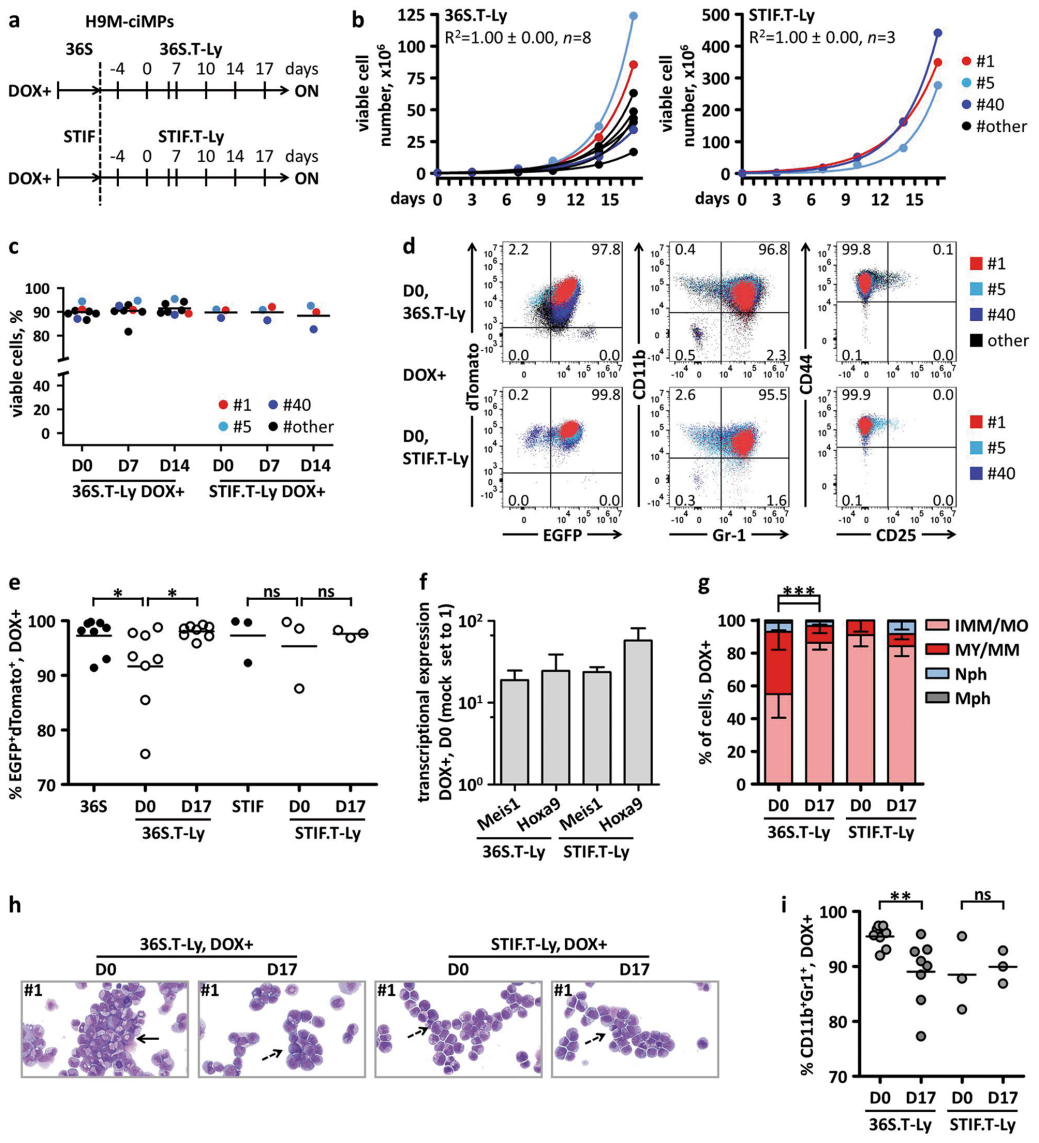
Characterisation of H9M-ciMPs cultured under T-lymphoid conditions and doxycycline application. (**a**) Experimental design describing the co-culture of H9M-ciMPs with OP9-DL1 stromal cells under T-lymphoid cytokine conditions following 36S (36S.T-Ly) and STIF (STIF.T-Ly) conditions, respectively, in the presence of 1 µg/mL doxycycline (DOX+ , ON). (**b**) Exponential growth equation for H9M-ciMPs under 36S.T-Ly and STIF.T-Ly conditions. R^2^, R-squared value. (**c**) Percentages of viable cell number under 36S.T-Ly (*n* = 8) and STIF.T-Ly (*n* = 3) conditions. (**d**) Dot plot overlays to present expression of EGFP^+^dTomato^+^, CD11b^+^Gr-1^+^ and CD44^+^CD25^+^ under 36S.T-Ly (*n* = 8) and STIF.T-Ly (*n* = 3) conditions (frequency percentages are given for H9M-ciMP #1). (**e**) Percentages of EGFP^+^dTomato^+^ under initial 36S and STIF conditions and under 36S.T-Ly (*n* = 8) and STIF.T-Ly (*n* = 3) conditions at D0/ON and D17/ON. D, day. (**f**) Histogram presentation of relative transcriptional expression of *Meis1* and *Hoxa9* (corresponds to *Hoxa9*-total) in H9M-ciMPs under 36S.T-Ly (*n* = 8) and STIF.T-Ly (*n* = 3) conditions. Expression levels in non-transduced non-cultured lineage negative cells (Mock) were set to 1. Data are represented as mean ± SD. (**g**) Histogram presentation of differential cell count for H9M-ciMPs under 36S.T-Ly (*n* = 8) and STIF.T-Ly (*n* = 3) conditions at D0/ON and D17/ON. IMM/MO, immature cells: myeloblast-, promyelocyte-, monoblast-, promonocyte- and monocyte-like cells; MY/MM, myelocyte-, metamyelocyte-like; Nph, band neutrophil, segmented neutrophil; Mph, macrophage. Data are represented as mean ± SD. ***, *P* < 0.001, statistically significant differences revealed for IMM/MO and MY/MM. (**h**) Cytospin analysis (May-Grünwald/Giemsa staining, magnification × 80) of selected cell line #1 for D0/ON and D17/ON. Dashed black arrows indicate immature myeloid cells; solid black arrows indicate more differentiated cells. (**i**) Percentages of CD11b^+^Gr-1^+^ cells in H9M-ciMPs cultured under 36S.T-Ly (*n* = 8) and STIF.T-Ly (*n* = 3) conditions at D0/ON and D17/ON. H9M-ciMPs, conditionally immortalised myeloid progenitor cell lines co-expressing *Hoxa9*/*Meis1.* H9M-ciMPs #1,5,40 are marked in red, light blue and blue, respectively, and #11,12,24,25,29 are marked in black as “others”. Each data point represents the result of an individual cell line. For (**c**, **e**, **i**), horizontal lines indicate mean values. ns, not significant, *p* > 0.05; *, *P* < 0.05; **, *P* < 0.01. Comparisons were made using non-parametric two-tailed Mann–Whitney test; (**c**) generated in Matplotlib 3.1.1 https://matplotlib.org; (**d**) generated in PowerPoint using plots from Flowjo 10 (Tree Star, Ashland, OR); (**e**, **i**) generated in Prism 5 (GraphPad Software, San Diego, CA); (**h**) generated in PowerPoint using pictures from NDP.view2.8.24 viewing software U12388-01, https://www.hamamatsu.com/eu/en/product/type/U12388-01/index.html.

Here, H9M-ciMP were cultured under DOX+ T-lymphoid (T-Ly: SCF, FLT3L, IL7) conditions^48^ using OP9-DL1 stromal cells^14,15^ following 36S (36S.T-Ly, *n* = 8) or STIF conditions (STIF.T-Ly, *n* = 3) (Fig. 6a). Surprisingly, under both 36S.T-Ly and STIF.T-Ly conditions, H9M-ciMPs revealed stable exponential growth (Fig. 6b). Survival under T-lymphoid conditions was confirmed by a high percentage of viable H9M-ciMPs, with 91.5% ± 2.6% (mean ± SD, *n* = 8) viability for 36S.T-Ly and 88.4% ± 5.1% (*n* = 3) viability for STIF.T-Ly conditions (D14) (Fig. 6c).

For 36S.T-Ly conditions, a slight initial decrease in the % of EGFP^+^dTomato^+^ cells (91.7% ± 7.8%, mean ± SD, *n* = 8) was observed at D0 (corresponds to 4 days of T-Ly pre-conditioning) (Fig. 6a,d,e). At D17, percentages of EGFP^+^dTomato^+^ cells increased to 98.1% ± 1.2% (mean ± SD, *n* = 8) indicating only initial adaptation to the new microenvironment (Fig. 6a,d,e). Interestingly, no significant adaptation drop in the % of EGFP^+^dTomato^+^ cells was detected for STIF.T-Ly treated H9M-ciMPs: 95.3% ± 6.7% for D0 and and 97.6% ± 0.7% for D17 (*n* = 3) (Fig. 6e).

For both 36S.T-Ly and STIF.T-Ly conditions, efficient *Hoxa9* and *Meis1* transcriptional upregulation was detected already at D0 (Fig. 6f).

In line with this, cytospin analysis of H9M-ciMPs cultured under both 36S.T-Ly and STIF.T-Ly conditions at D0 and D17 revealed blockade of myelomonocytic differentiation, but with a transient increase in the percentage of more differentiated myelocytes and metamyelocytes for 36S.T-Ly at D0 in comparison with D17 (Fig. 6g,h).

Although a statistically significant difference in the percentage of CD11b^+^Gr-1^+^ cells was observed between D0 and D17 for 36S.T-Ly (Fig. 6i), the expression pattern of CD44/CD25 markers did not change over this time (Fig. 6d; Supplementary Fig. S10a). Thus, both 36S.T-Ly and STIF.T-Ly cultured H9M-ciMPs were ~ 100% CD44^+^CD25^–^ on D0 (99.4% ± 0.5%, mean ± SD, *n* = 8; 99.4% ± 0.5%, *n* = 3, respectively) (Fig. 6d). On D17, H9M-ciMPs CD44/CD25 expression pattern was similar (36S.T-Ly: 98.8% ± 1.4%, mean ± SD, *n* = 8; STIF.T-Ly: 99.3% ± 0.2%, *n* = 3), demonstrating efficient support of myeloid cells under T-lymphoid microenvironment conditions as reported recently^48^ (Supplementary Fig. S10a).

Importantly, mock non-transduced Lin^−^ cells already showed T-lymphoid differentiation at D17, with percentages of cells in the double-negative (DN) stages corresponding to previously described in vitro assays^48^: DN1, 3.1%; DN2, 13.2%; DN3, 73.5%; and DN4, 10.2% (Supplementary Fig. S10b).

Investigation of the transcriptional expression of Notch signalling genes in H9M-ciMPs kept under 36S.T-Ly or STIF.T-Ly conditions in the presence of DOX showed transcriptional upregulation of Notch receptor *Notch3* and Notch ligand *Jag1* (Supplementary Fig. S11a). Mock non-transduced non-cultured Lin^−^ cells were selected as reference material. Notch target *Hes1*, which plays a role in stemness, metastasis and multidrug resistance^49,50^, was also upregulated (Supplementary Fig. S11a). Interestingly, for H9M-ciMPs cultured under 36S and STIF conditions (Figs. 2a; 3b), *Notch3* and *Hes1* were either down-regulated or unchanged (Supplementary Fig. S11b).

Although the role of *Notch3* is poorly understood^51,52^, recent studies using a zebrafish model suggested that *Notch3* is required to activate Notch signalling, and this activation is necessary for further HSC specification^52^. Thus, *Notch3* up-regulation in H9M-ciMPs under T-Ly conditions might be considered as a part of adaptative H9M-ciMP properties.

Interestingly, after DOX removal, H9M-ciMPs cultured under 36S.T-Ly or STIF.T-Ly did not show any significant increase in viable cell number after DOX withdrawal (*P* > 0.05), indicating myeloid differentiation and depletion after DOX removal (Supplementary Fig. S12). Of note, a similar observation was obtained for STIF conditions after DOX removal (Supplementary Fig. S12).

To conclude, H9M-ciMPs revealed efficient adaptation to T-Lymphoid conditions. For H9M-ciMPs pre-cultured in STIF (STIF.T-Ly), exponential growth under T-Ly conditions was more remarkable.

## Discussion

Tetracycline-regulated models for genetic modifications of HSCs help explore the role of collaborating and single genes in complex signalling networks that control the fate decision process in normal and malignant haematopoiesis. Improved understanding of immortalised cell growth behaviour, mechanisms that trigger differentiation and microenvironmental influences on these processes could help develop optimised strategies for HSC ex vivo manipulation and leukaemia treatment.

Here, we present an inducible model based on tetracycline-regulated co-expression of *Hoxa9* and *Meis1* in murine HPCs potentially collaborating with genes targeted via insertional mutagenesis and showing myelomonocytic arrest under different cytokine/microenvironmental conditions.

The vector design and drug-dependent selection approach allowed us to isolate H9M-ciMP cell lines with DOX-dependent exponential growth under myelomonocytic^2^, stem cell maintaining^13^ and T-lymphoid conditions^14,15,48^. Limiting dilution assays to select H9M-ciMPs in the presence or absence of DOX demonstrated that MRV integration without simultaneous *Hoxa9/Meis1* co-expression did not lead to the efficient selection of long-term proliferating cell lines.

The insertional analysis identified a number of potential genes involved in cancerogenesis, immune response, differentiation and metabolism, which may potentially cooperate with *Hoxa9/Meis1* in cell line establishment (Supplementary Table S2). Hypothesising that insertional dysregulation of these genes could contribute to H9M-ciMP survival/differentiation under suboptimal intrinsic and extrinsic conditions, we initially focused on eight H9M-ciMP cell lines that had RVIs in the loci of *Ninj2*^12^ (#1, 5), *Vps45/Otud7b*^25,26^ (#11), *Plet1*^27^/*Thada*^28^ (#12), *Cmah*^24^ (#24, 25, 29) and *Chsy1*^29^ (#40) (Supplementary Table S1, S2).

Investigation of transgene modulation (i.e. ON/OFF/re-ON experiments) under myelomonocytic conditions revealed selective H9M-ciMP survival and reversibility. Thus, five H9M-ciMP cell lines with RVIs into loci of *Ninj2*^12^ (#1, 5), *Vps45*/*Otud7b*^25,26^ (#11), *Plet1*^27^ (#12) or *Chsy1*^29^ (#40) showed rapid restoration of exponential growth after DOX reapplication, which was related to slower differentiation after DOX removal and probably longer LIC preservation.

Genes surrounding the corresponding RVI loci (window ~  ± 250 Kb) in “reversible” cell lines were shown to be involved in haematological disorders or solid tumour proliferation. For instance, enhanced *NINJ2* expression was reported to be associated with enhanced growth, survival and proliferation of human glioma and colorectal cancer cells^36,37^. The epigenetic regulator *Kdm5a*^44^, a member of the gene group, often mutated in myelodysplastic syndrome (MDS) (Supplementary Table S2), was found to be one of the genes within ~  ± 250 Kb from the RVI in the *Ninj2* locus. Mutations in *Vps45* are associated with congenital neutrophil defect syndrome and impaired neutrophil function^25^. *Chsy1* may have a role in the generation and maintenance of neutrophils and macrophages, involved in neurodegenerative processes and proliferation promotion in colorectal cancers^29,53^.

Importantly, three H9M-ciMP cell lines with identical RVI into the *Cmah* locus (#24, 25, 29) rapidly differentiated towards neutrophils/macrophages and were incapable of reversibility after DOX removal.

The “reversible” H9M-ciMP cell lines #1 and 5, which had identical RVI into the *Ninj2* locus, demonstrated exponential proliferation under stem cell maintaining^13^ conditions and were selected for serial murine transplantation. The LIC potential of these cell lines was verified by the rapid development of DOX-dependent AMLs and verification of the RVI in the *Ninj2* intron 1 in bone marrow cells. The immunophenotype and morphology of immature myelomonocytes co-expressing *Meis1* and *Hoxa9* and infiltrating haematopoietic organs did not significantly depend on cytokine conditions used before transplantation (myelomonocytic or stem cell maintaining).

Interestingly, while myelomonocytic conditions are commonly used in murine transplantation models and leading to AML^7^, stem cell maintaining conditions were described to contribute to lymphoid leukaemia development^34^. The latter was described even for shorter exposure of retrovirally transduced HPCs to STIF conditions than in the present experiment^34^.

We characterised the transcriptional expression of the following genes within of ~  ± 250 Kb from RVI in the affected *Ninj2* locus: *Ninj2*, *Kdm5a*, *Rad52* and *Wnk1.* RVI into the 1st intron of *Ninj2* resulted in its transcriptional down-regulation when compared to non-transplanted DOX-treated murine cells. Significantly, *NINJ2* is also downregulated in human AMLs according to the integrated BloodSpot database^46^. Interestingly, the dysregulation level of other investigated genes was not directly related to gene location regarding of RVI. Thus, more distal *Kdm5a* and *Rad52* (~ 240 Kb upstream and ~ 213 Kb downstream of RVI, respectively) were down-regulated, while *Wnk1* (~ 77 Kb upstream) expression remained similar when compared to non-transplanted DOX-treated murine cells. However, the orientation of the retroviral vector sequence may be important as the provirus was in the forward orientation for all down-regulated transcriptional units (Supplementary Table S2).

Of note, in contrast to selective survival of H9M-ciMPs under STIF conditions, all investigated H9M-ciMP cell lines survived in T-lymphoid conditions after a short adaptation period. Thus, cell lines co-expressing *Hoxa9 and Meis1* and possessing different RVIs demonstrated the exponential growth of immature myelomonocytic cells. Therefore, we suggest that co-expression of *Hoxa9* and *Meis1* is necessary for microenvironmental adaptation properties ex vivo and maintaining myelomonocytic memory.

**Figure 7:**
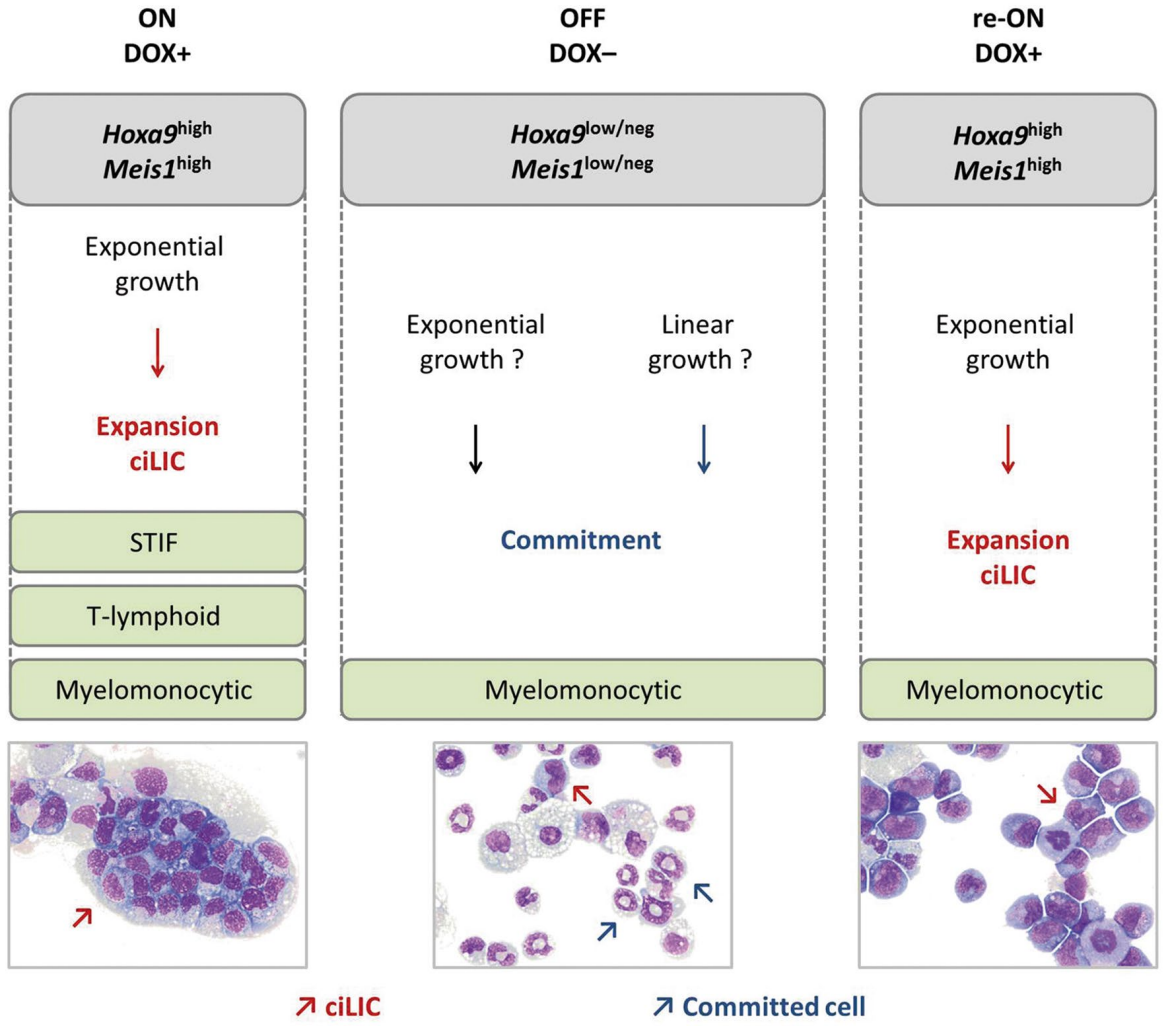
ciLICs co-expressing *Hoxa9/Meis1* under different microenvironmental conditions ex vivo upon switching doxycycline ON/OFF/re-ON. Doxycycline (DOX) application (DOX+ , ON), removal (DOX-, OFF) reapplication (DOX+ , re-ON); ciLIC, conditionally immortalised leukaemia initiating cells; STIF, stem cell maintaining conditions; T-lymphoid, co-culture with OP9-DL1 stromal cells under T-lymphoid conditions; neg, negative*.* Red arrows indicate ciLICs; blue arrows indicate committed cells, e.g. neutrophils.

Myelomonocytic conditions initially used to generate H9M-ciMPs defined the myelomonocytic immature signature kept under both stem cell maintaining and T-lymphoid conditions in the presence of DOX. Our results are in accordance with previous findings that demonstrated myeloid differentiation to be critical for LIC formation and AML development^54^. Together with the possible RVI influence, this suggests a unique role of myelomonocytic conditions for H9M-ciMP survival (Fig. 7).

A limitation of the current study is that AML in vivo data were obtained for only two conditionally immortalised H9M-ciMP cell lines characterised by identical RVI in the *Ninj2* locus, indicating that they may have originated from a common ancestor. Thus, additional in vivo experiments are needed to test LIC capacities of other H9M-ciMPs that have other RVIs. Obtained fate differences depending on RVI present in a given H9M-ciMP cell line and the direct implication of IM targeted genes could be of particular interest in future experiments. This system may also be useful to study if H9M-ciMPs can maintain LIC ability in vivo after culturing under T-lymphoid conditions. In summary, the ciLIC system co-expressing *Hoxa9* and *Meis1* provides a promising tool to study LIC properties.

## Methods

### Vector construction

The TRP T11^10^ was inserted into the 3′ LTR of the self-inactivating gammaretroviral vector^9^ (Fig. 1a). No linker (MRV*) or a 304 bp linker (MRV) was cloned in front of the T11 TRP. Such linker provides an additional sequence for LM-PCR primer design to investigate insertion sites. Both vector versions were used in two independent experiments for H9M-ciMP generation. The coding DNA for murine *Hoxa9*^1^ was located under the control of mPGK promoter in the constitutive module of the vector. Correspondently, coding DNA for murine *Meis1*^1^ was cloned into the inducible module of the vector resulting in construct RSF91.Meis1.idTomato.mPGK.Hoxa9.P2a.EGFP.pre.T11 (Fig. 1a). To track the *Hoxa9* expression, *EGFP* was linked by 2A self-cleavage sites^1^. To track the *Meis1*, we introduced the red fluorescent protein dTomato via an internal ribosomal entry sequence (IRES) derived from encephalomyocarditis virus downstream of the reading frame. Vectors and cloning details are available on request.

### Vector production

Vector production and titration was performed as described before^2,55^. In short, 293T cells were transfected with 10 µg of retroviral vector, 3 µg of pcDNA3.MLVsyn g/p, 2 µg of pMD.G (VSVg) using the calcium phosphate method. Viral supernatant was concentrated by ultracentrifugation and titrated using the SC1-rtTA2 cell line as described before^2^.

### H9M-ciMP generation and cultivation under the myelomonocytic and stem cell maintaining conditions

Lin^−^ cells were magnetically sorted from BM using lineage-specific antibodies (Lineage Cell Depletion Kit, Miltenyi Biotech, Bergisch Gladbach, Germany). To generate H9M-ciMPs, Lin^−^ cells were prestimulated for 24 h and transduced overnight with multiplicities of infection 10 in the absence of doxycycline in serum-free StemSpan medium (Stem Cell Technologies, Vancouver, Canada) supplemented with 1% penicillin/streptomycin (Pan Biotech), 2 mM l-glutamine (Biochrom, Berlin, Germany), as described before using myelomonocytic conditions 36S: 6 ng/mL of murine interleukin 3 (mIL3); 10 ng/mL of human interleukin 6 (hIL6); 20 ng/mL of murine stem cell factor (mSCF) (all cytokines from Peprotech, Hamburg, Germany)^1,2,7^. Doxycycline Hyclate (DOX) (Sigma-Aldrich, St. Louis, MO) was added (1.0 µg/mL) 1 day after transduction to half of the transduced cells. Another half was kept in the DOX absence as the negative control. On days 13–23 post-transduction, HOXA9.EGFP^+^/MEIS1.dTomato^+^ cells and HOXA9.EGFP^+^/MEIS1.dTomato^−^ kept under DOX + and DOX- conditions respectively were sorted on a FACSAria Fusion instrument by the Hannover Medical School (MHH) Cell Sorting Unit. Limiting dilution assay was performed by seeding 1 (MRV*) or 10 (MRV) cells per well in 96 well plates. Limiting dilution assay of sorted HOXA9.EGFP^+^/MEIS1.dTomato^−^ cells (kept before sorter under DOX- conditions) was performed under DOX- (MRV*) and DOX+ (MRV) conditions, and did not result in the selection of stably proliferating cell lines. Only H9M-ciMPs kept under DOX+ conditions before sorting and under limiting dilution assay (MRV* and MRV) were cryopreserved on day 60–85 post-transduction. H9M-ciMPs were generated in two independent experiments performed in three biological replicates for DOX+ conditions and two biological replicates for DOX- conditions.

After sorting/cryopreservation, H9M-ciMPs were kept in Iscove's Modified Dulbecco's medium (Biochrom) supplemented with 15% fetal bovine serum Brazil One (FBS; Pan Biotech, Aidenbach, Germany), 1% penicillin/streptomycin, 2 mM l-glutamine, using myelomonocytic (36S) or stem cell maintaining cytokine conditions, STIF: 50 ng/mL of mSCF, 20 ng/mL of murine thrombopoietin (mTPO), 20 ng/mL of murine insulin-like growth factor 2 (mIGF2), 10 ng/mL of human fibroblast growth factor 1 (hFGF1) (all cytokines from Peprotech) with 10 µg/mL of heparin (Sigma-Aldrich)^13,56^.

### T-lymphoid conditions using OP9-DL1 co-culture assay

OP9-DL1^14,48^ cells were cultured in alpha minimum essential medium (α-MEM) freshly reconstituted from a powder (Gibco, Paisley, Scotland, United Kingdom) supplemented with 20% FBS Brazil One (Pan Biotech, Aidenbach, Germany), 1% penicillin/streptomycin, and 2.2 g/L sodium bicarbonate (Pan Biotech). H9M-ciMPs were transferred to OP9-DL1 feeder layer in complete α-MEM supplemented with 20 ng/mL of mSCF (Peprotech), 5 ng/mL of human FMS-like tyrosine kinase 3 ligand (hFLT3L; Peprotech), and 2 ng/mL of human interleukin 7 (hIL7; Cytheris S.A., Issy Les Moulineaux, France) and cultured as described before^48^.

### Flow cytometry

Cells were stained with the following anti-mouse antibodies: CD11b APC-Cy7 (M1/70), Gr-1 PE-Cy7 (RB6-8C5 ), CD44 BV711 (IM7), CD25 APC (PC61) (all from Biolegend, San Diego, CA); c-KIT APC (2B8) and B220 PerCP-Cy5.5 (RA3.6B2) (both from eBioscience, San Diego, CA). Sample acquisition was performed using CytoFLEX S (Beckman Coulter Life Sciences, Brea, CA) or FACSCalibur (Becton Dickinson, Heidelberg, Germany) flow cytometers. DAPI (4′,6-diamidine-2′-phenylindole dihydrochloride; Sigma-Aldrich) or PI (propidium iodide; Sigma) were used to label dead cells. The data were analysed with FlowJo 10 software (Tree Star, Ashland, OR).

### Mice and transplantation conditions

C57BL/6J mice were purchased from the Hannover Medical School (MHH) central animal facility (Hannover, Germany) and Charles River (Sulzfeld, Germany). Rosa26rtTA-nls-Neo2 (Rosa26rtTA) mice expressing the reverse tetracycline-inducible transactivator (*rtTA2*) under the control of the ubiquitously active Rosa26 locus, were purchased from the central MHH animal facility (Hannover, Germany)^16–18^. Both C57BL/6J and Rosa26rtTA mice were housed in micro-isolators under pathogen-free conditions in MHH animal facility. Rosa26rtTA mice (female, age 16 (MRV) or 28 (MRV*) weeks) served as the source of Lin^−^ cells for immortalisation setup ex vivo (see H9M-ciMP generation). For the in vivo 1° transplantation assay, H9M-ciMPs #1 and #5 cultured in 36S (Fig. 1b,d) or STIF (Fig. 3a) cytokine conditions were pooled accordingly (Supplementary Table S1). 0.8 × 10^6^ (36S, ~ month 4) or 0.7 × 10^6^ (STIF, day 23) viable H9M-ciMPs were transplanted intravenously per 1° recipient C57BL/6J (female, age 10 weeks) after conditioning by myeloablative irradiation (9 Gy) (Supplementary Table S1). 3 × 10^5^ freshly isolated total bone marrow cells were co-transplanted per 1° recipient for radioprotection. For in vivo 2° transplantation assay, 1.8 × 10^6^ cells of the selected 1° recipient were transplanted intravenously per 2° recipient (age 13 weeks) after 9 Gy irradiation (Supplementary Table S1). For doxycycline administration, 1° and 2° recipients received standard dry food supplemented with doxycycline hyclate (Sigma-Aldrich) at a final concentration 625 mg/kg (Ssniff, Soest, Germany), starting one week before transplantation and throughout the experiment^56^. All transplanted mice received 100 µg/ml of ciprofloxacin (Fresenius Kabi, Bad Homburg, Germany) within the drinking water for the first two weeks. The animal experiment protocol (TS11/0607) was approved by MHH’s central animal facility (Hannover, Germany) and Lower Saxony State Office for Consumer Protection and Food Safety [LAVES] (Lower Saxony, Germany), which includes an ethical review. All experiments were performed in accordance with their guidelines and regulations. As soon as symptoms of myeloproliferative disease or acute leukaemia were detected, the animals were immediately euthanised and analysed in detail. The animal experiments were carried out in compliance with the following ARRIVE guidelines (https://arriveguidelines.org/arrive-guidelines) Essential 10: study design, sample size, inclusion and exclusion criteria, randomisation, blinding, outcome measures, statistical methods, experimental animals, experimental procedures, and results.

### Analysis of leukaemias

Peripheral blood cells were taken by retro-orbital bleeding and measured using an automatic analyser (ABC Counter, Scil, Vierheim, Germany). Mice were euthanised when symptomatic and macroscopically examined for pathological abnormalities. Enlarged organs were weighed. Bone marrow, spleen, lymph nodes and thymus cell suspensions were prepared for cytospins and flow cytometry analysis as described before^1,56^.

### VCN determination

qPCR to determine the VCNs was performed on an Applied Biosystems StepOnePlus System (Foster City, CA) in triplicates using the Quantitect SYBR Green Kit (Qiagen, Hilden, Germany) as described before^1^. Briefly, a plasmid standard containing the sequences of woodchuck hepatitis virus post-transcriptional regulatory element (wPRE) and fetal liver kinase 1 (Flk1) gene as internal reference control was used for quantification with the described primer sets^1^.

### RT-qPCR

RT-qPCR was performed as described before^1^. RT-qPCR analysis was performed on an Applied Biosystems StepOnePlus System in triplicates using the Quantitect SYBR Green Kit (Qiagen). Relative quantification of a target gene transcript in comparison with a reference β-actin transcript^1^ was performed using the method described by Pfaffl^57^. The program https://qpcr.probefinder.com/organism.jsp was used to design primers. RT-qPCR efficiencies were determined for all sets of designed primers^57^. Sequences of primers used: Hoxa9 forward (Fw), 5′- TCCCTGACTGACTATGCTTGTG-3′; Hoxa9 reverse (Rv), 5′-GTTGGCAGCCGGGTTATT-3′; Meis1 Fw, 5′-GACGCTTTAAAGAGAGATAAAGATGC-3′; Meis1 Rv, 5′- CATTTCTCAAAAATCAGTGCTAAGA-3′; Ninj2 Fw, 5′-CAGGACCTCCAGCAATCCTA-3′; Ninj2 Rv, 5′-TCAGGGACAAAGGCTGAAGT-3′; Wnk1 Fw 5′-GCAGCATCTTCCAGTGAAGG-3′; Wnk1 Rv, 5′-GCTACTGTCTGGGAAACTGGA-3′; Rad52 Fw, 5′-CCCCTTTTTCAGACATCACC-3′; Rad52 Rv, 5′-CCATCCACTGAAGCCAAAGTA-3′; Kdm5a Fw, 5′-GGAAGGACTGGAGGAGTCAA-3′; Kdm5a Rv, 5′-TCCTTTTGCTCTCGTTCTTTG-3′.

### Identification of integration sites

Integration site analysis was performed by LM-PCR as reported before^11,58^. Genomic DNA was digested with CviQI restriction enzyme for MRV and with HaeIII for MRV* pre-selected H9M-ciMPs (New England BioLabs, Frankfurt/ Main, Germany). To amplify integration sites of MRV vectors, 5′ LM-PCR was performed using the following primers Rv1 5′-[bio] ACGTTCTCTATCACTGATAGGGAGTAAACTGG-3′; Rv2, 5′-CAGGCACAAGTGTTAAAGCAG-3′; Rv3, 5′-TCGAATTCACGTGTCGACGA-3′. To amplify integration sites of MRV* vectors, 3′ LM-PCR was performed using a primer that annealed in the wPRE sequence (SINPRE, 5′-[bio]GCACTGATAATTCCGTGGTGTTGTC-3′). For exponential PCR, the following primers were designed: SIN LTR2_1, 5′-GTTTGGCAAGCTAGCGAGACT-3′ and/or SIN LTR2_2, 5′-CCCTATCAGTGATAGAGAACGT-3′; SIN LTR3, 5′-CCCAATAAAGCCTCTTGCTGT-3′. PCR products were extracted after gel electrophoresis using QIA quick Gel Extraction Kit (Qiagen), sequenced and analysed using BLAST/BLAT searches at www.ensembl.org/Mus_musculus/Tools/Blast42 (Ensembl release 100 April 2020)^59^. LM-PCR for H9M-ciMP #38 did not reveal any specific bands. The images of full-length gels presented in Supplementary Figure S1, S9. The photos were taken using BIO-RAD Molecular Imager Gel Doc XR + System with Image Lab Software (Bio-Rad Laboratories, Inc. Hercules, California, USA).

### Cytospin analysis

2 × 10^4^ − 2 × 10^5^ cells were centrifuged in 150 µl PBS for 10 min at 800 rpm on microscope slides using a Thermo Shandon Cytospin 4 centrifuge (Thermo Fisher Scientific, Waltham, MA, USA). Cytospins were stained after Pappenheim (Giemsa/May-Grünwald): 5 min in May-Grünwald followed by 20 min in Giemsa staining solutions (Sigma-Aldrich, Steinheim, Germany). The cytospins were digitised at a 40 × magnification using the Hamamatsu NDP.scan software (version 3.2.17) with the Hamamatsu NanoZoomer S210 digital slide scanner equipped with a Nikon plan apochromat 20x (NA 0.75) objective lens and a colour CMOS sensor type 1/2.3 camera (pixel 4000 × 3000). Morphology was assessed using NDP.view 2.8.24 viewing software U12388-01 https://www.hamamatsu.com/eu/en/product/type/U12388-01/index.html by examination of digitised cytospins; a 90–200-cell differential count was performed. Any cell which did not fit a definition exactly was counted with the category, which it most closely resembled^32^. Representative pictures of digitised cytospins or light microscope pictures of cytospins (BX51 microscope, camera XC50, software Cell F version 3.4, all Olympus) are presented.

### Statistical analysis

Ex vivo results were presented as the mean of individual parameters obtained from different H9M-ciMP cell lines. Animal experiment results were presented as the median for spleen weight and WBC count and as the mean for other results. Statistical analyses were performed using GraphPad Prism versions 4.00 or 5.00 for Windows (GraphPad software, San Diego, CA) or Matplotlib 3.1.1 https://matplotlib.org^48,60^. Comparisons were made using the non-parametric two-tailed Mann–Whitney test. *P-*values < 0.05 were considered statistically significant: *, *P* < 0.05; **, *P* < 0.01; ***, *P* < 0.001; ****, *P* < 0.0001.

## Supplementary Information

Supplementary Information

## Data Availability

The datasets generated and/or analysed during the current study are available from the corresponding authors on reasonable request.
