# Supplementary material for: Conditionally immortalised leukaemia initiating cells co-expressing Hoxa9/Meis1 demonstrate microenvironmental adaptation properties ex vivo while maintaining myelomonocytic memory
Source: Sci Rep. 2021 Mar 5;11:5294. doi: 10.1038/s41598-021-84468-3 (PMC7935976; doi:10.1038/s41598-021-84468-3)
Supplement: Supplementary file 1 — Supplementary Information [file 41598_2021_84468_MOESM1_ESM.pdf]

Supplementary information:

Title: Conditionally immortalised leukaemia initiating cells co-expressing *Hoxa9/Meis1* demonstrate microenvironmental adaptation properties *ex vivo* while maintaining myelomonocytic memory.

Author list:
Maike Stahlhut, Teng Cheong Ha, Ekaterina Takmakova, Michael A. Morgan, Adrian Schwarzer, Dirk Schaudien, Matthias Eder, Axel Schambach*, Olga S. Kustikova*

*Co-corresponding authors

Supplementary Figure S1

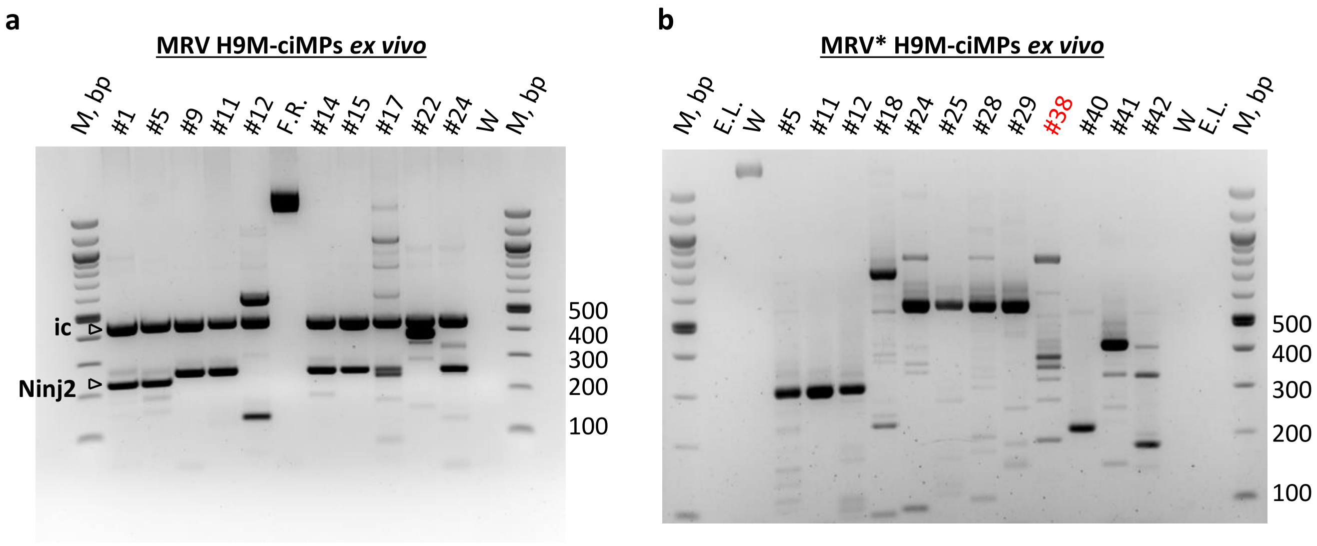

Supplementary Figure S1. Insertional analysis of H9M-ciMP cell lines cultured *ex vivo* under myelomonocytic conditions. (a) Ligation-mediated PCR (LM-PCR) analyses of MRV H9M-ciMPs to identify insertion sites. **(b)** LM-PCR analyses of MRV* H9M-ciMPs to identify insertion sites. D, day; DOX+, 1 µg/mL doxycycline. F.R., fail of LM-PCR reaction. MRV, multimodal retroviral vector; MRV*, multimodal retroviral vector without adapter for LM-PCR. W, water; M, 100 bp marker; bp, base pair; ic, internal control. #38 in red: for #38 no specific band/insertion was obtained. H9M-ciMPs, conditionally immortalised myeloid progenitor cell lines engineered to co-express *Hoxa*9 and *Meis1*.

Supplementary Figure S2

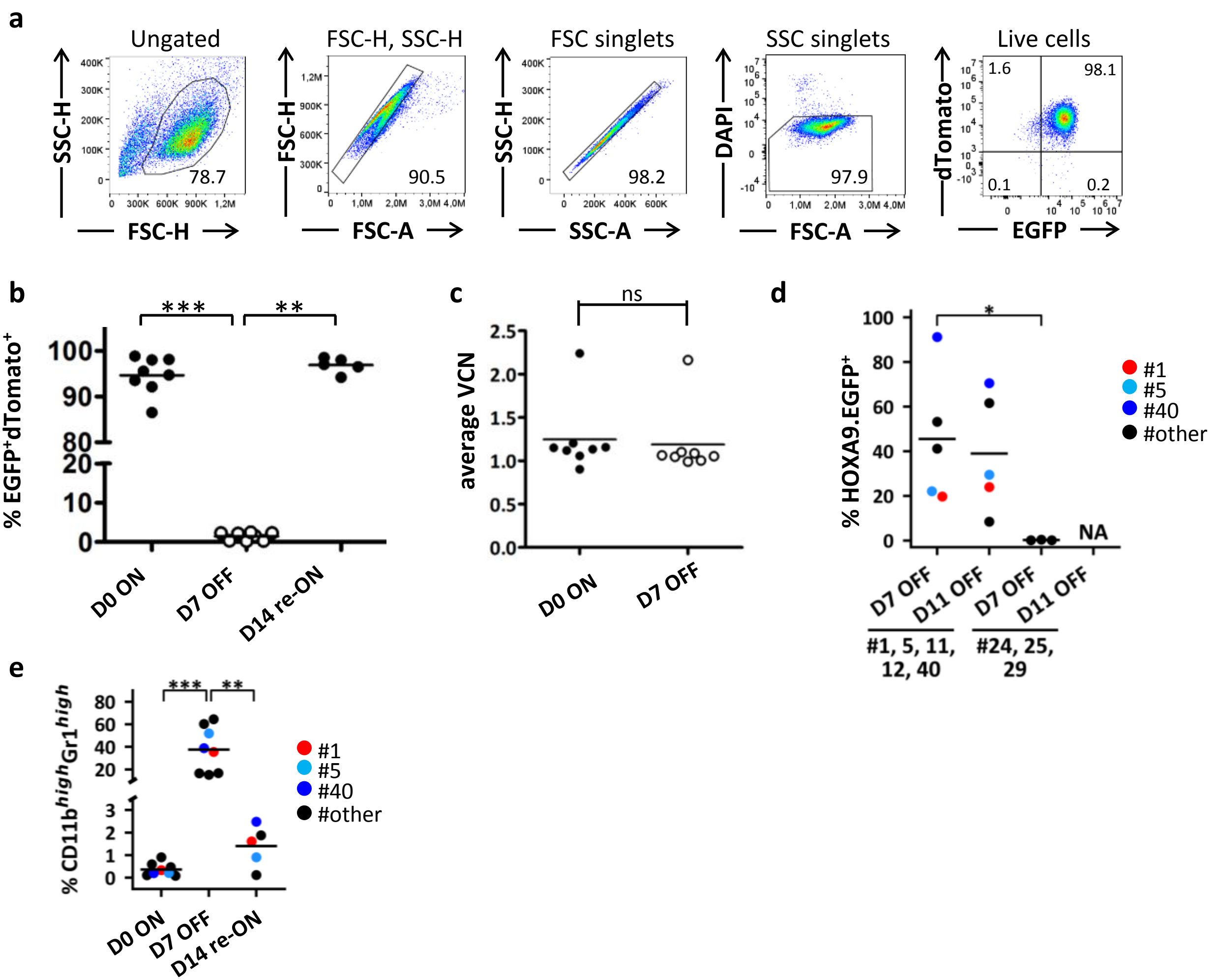

Supplementary Figure S2. Characterisation of H9M-ciMPs under myelomonocytic conditions, doxycycline ON/OFF/re-ON. **(a)** Gating strategy of flow cytometric analysis using a CytoFLEX cytometer (presented for myelomonocytic (36S) conditions). Cell populations were gated on forward scatter (FSC) and side scatter (SSC) and, after doublet exclusion, gated for viable 4′,6-diamidino-2-phenylindole (DAPI) negative cells. **(b)** Percentages of EGFP^{+}dTomato^{+} cells under D0/ON, D7/OFF 36S conditions (*n*=8) and D14/re-ON (*n* =5) 36S conditions. **(c)** Average vector copy number (VCN) determined for D0/ON and D7/OFF conditions (*n*=8). **(d)** Percentages of HOXA9.EGFP+ expression after DOX removal at D7/OFF and D11/OFF. **(e)** Expression of CD11b^{high}Gr-1^{high} in H9M-ciMPs at D0/ON, D7/OFF, D14/re-ON. H9M-ciMPs, conditionally immortalised myeloid progenitor cell lines engineered to co-express *Hoxa*9 and *Meis1*. D, day; ON, 1 µg/mL DOX; OFF, DOX removal; re-ON, reapplication of 1 µg/mL DOX. H9M-ciMPs #1,5,40 are marked in red, light blue, blue, respectively, and #11,12,24,25,29 are marked in black as “others”. Each data point represents the result of an individual cell line. Horizontal lines represent the mean values. ns, not significant, P>0.05; *, P<0.05; **, P<0.01; ***, P<0.001 (non-parametric two-tailed Mann-Whitney test). NA, not analysed due to low cell number. H9M-ciMPs, conditionally immortalised myeloid progenitor cell lines engineered to co-express *Hoxa*9 and *Meis1*. DOX, doxycycline. Fig. S2b,c, generated in Prism 5 (GraphPad Software, San Diego, CA), Fig. S2d,e, generated in Matplotlib 3.1.1 https://matplotlib.org

Supplementary Figure S3

a

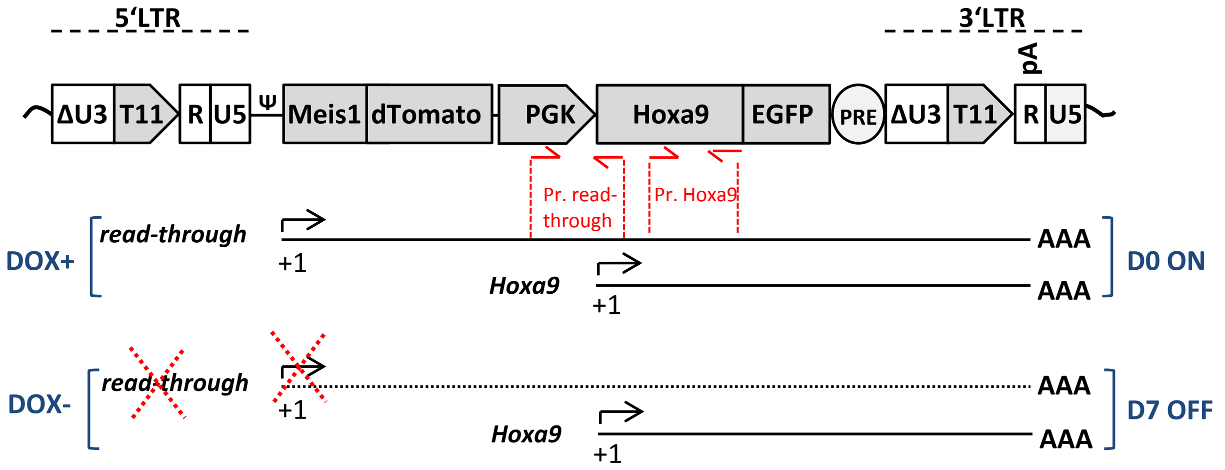

b

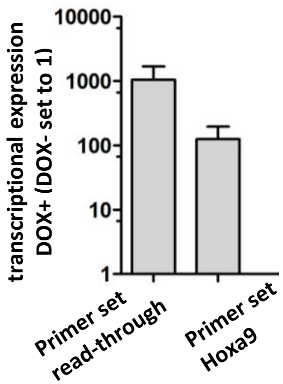

Supplementary Figure S3. "Read-through" transcription under doxycycline (DOX) supplementation impact the level of *Hoxa9* transcription when compared to DOX removal conditions. (a) Schematic presentation of integrated provirus to enable constitutive Hoxa9.EGFP and inducible Meis1.dTomato gene co-expression and primer design used for transcriptional analysis. Δ, SIN configuration with partially deleted U3 of the 3′ long terminal repeat (LTR); ψ, packaging signal; PRE, post-transcriptional regulatory element; EGFP, dTomato, fluorescent proteins. +1, transcriptional start site; pA, polyadenylation signal; arrow indicates primer design for RT-qPCR. **(b)** Histogram presentation of relative transcriptional expression of "read-through" transcript and "Hoxa9" transcript. Expression levels in the DOX absence were set to 1. Data are represented as mean ± SD, *n*=3. Pr., primer set. D, day; D0 ON, 1 µg/mL DOX (DOX+); D7 OFF, no DOX (DOX-).

Supplementary Figure S4

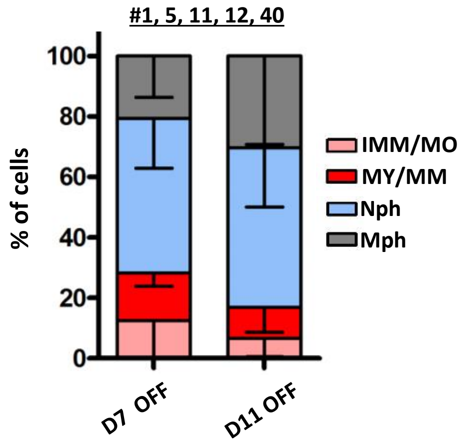

Supplementary Figure S4. Histogram presentation of differential cell count after doxycycline removal (OFF) for H9M-ciMPs #1, 5, 11, 12, 40 under myelomonocytic conditions. IMM/MO, immature cells: myeloblast-, promyelocyte-, monoblast-, promonocyte- and monocyte-like cells; MY/MM, myelocyte-, metamyelocyte-like; Nph, band neutrophil, segmented neutrophil; Mph, macrophage. Data are represented as mean ± SD, *n*=5. H9M-ciMPs, conditionally immortalised myeloid progenitor cell lines engineered to co-express *Hoxa*9 and *Meis1*. D, day.

Supplementary Figure S5

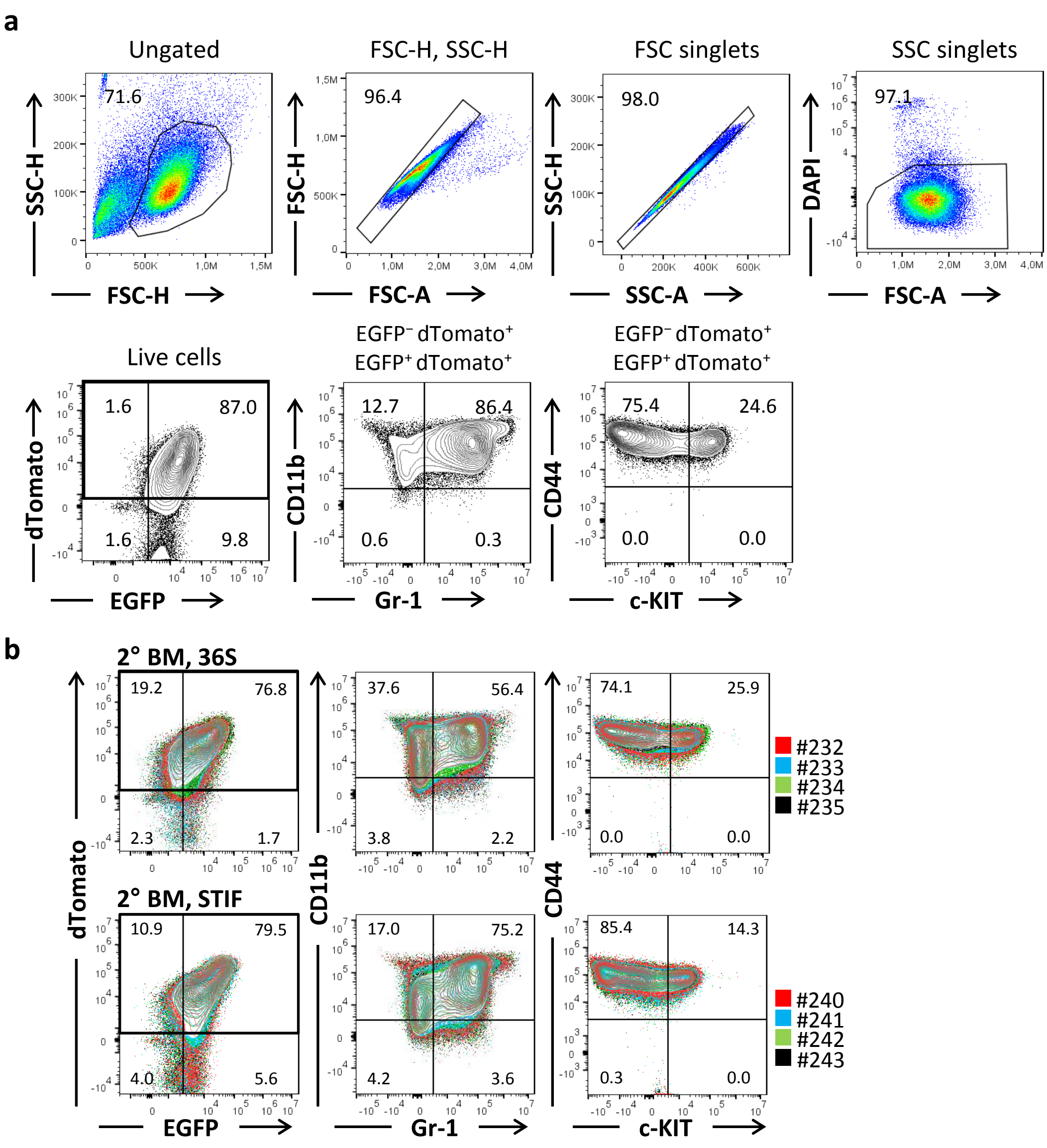

Supplementary Figure S5. Gating strategy and immunophenotype analysis of bone marrow cells from doxycycline-dependent H9M-ciMP induced AMLs. (a) Gating strategy of flow cytometric analysis using a CytoFLEX cytometer (presented for primary (1°) bone marrow (BM) cells of mouse #221). Cell populations were gated on forward scatter (FSC) and side scatter (SSC) and, after doublet exclusion, gated on viable 4′,6-diamidino-2-phenylindole (DAPI) negative cells. After gating for viable cells, target cell populations were gated for EGFP^{-}dTomato^{+} and EGFP^{+}dTomato^{+} cells followed by analysis for expression of CD11b/Gr-1 and other markers. **(b)** Immunophenotype analysis of BM cells from doxycycline-treated secondary (2°) recipients transplanted with BM from 1° recipients originally transplanted with H9M-ciMPs cultured under myelomonocytic (36S) or stem cell maintaining (STIF) conditions. Flow cytometry plot overlays to present expression of CD11b/Gr-1 and CD44/c-KIT markers in the EGFP^{+}dTomato^{+} and EGFP^{-}dTomato^{+} cells. Frequency percentages are given for recipients #235 (36S) and #243 (STIF). H9M-ciMP, conditionally immortalised myeloid progenitor cell lines engineered to co-express *Hoxa9* and *Meis1*. AML, acute myeloid leukaemia.

Supplementary Figure S6

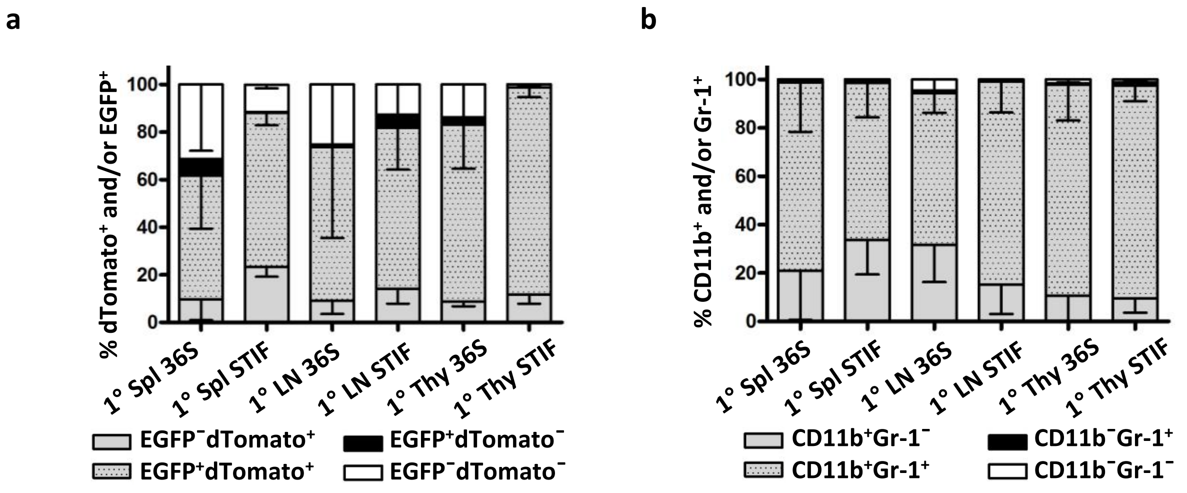

Supplementary Figure S6. Histogram presentation of transgene marking and expression of CD11b and/or Gr-1 in the cells from the spleen, lymph nodes and thymus from primary doxycycline-dependent H9M-ciMP induced AMLs. (a) Percentages of EGFP and/or dTomato expression in the spleen (Spl), lymph node (LN) and thymus (Thy) cells of primary (1°) 36S and STIF recipients. **(b)** Expression of CD11b and/or Gr-1 (gated on $EGFP^+dTomato^+$ and $EGFP^-dTomato^+$ cells) in 1° 36S and STIF recipients. Data are represented as mean ± SD for animals in each group, *n*=4 (1° STIF LN and Thy *n*=3). 36S, myelomonocytic conditions; STIF, stem cell maintaining conditions; AML, acute myeloid leukaemia. H9M-ciMPs, conditionally immortalised myeloid progenitor cell lines engineered to co-express *Hoxa9* and *Meis1*.

Supplementary Figure S7

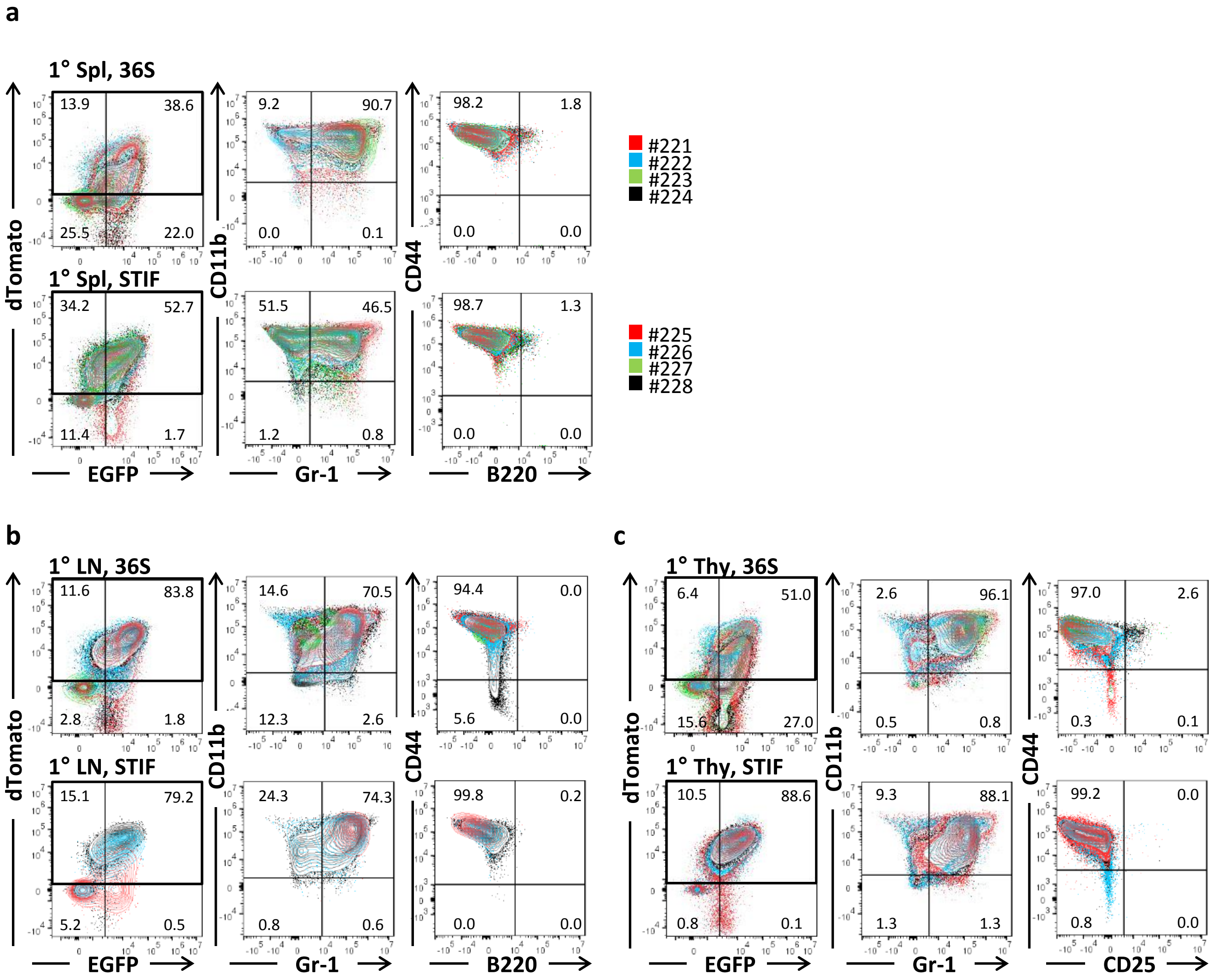

Supplementary Figure S7. Flow cytometric analysis of spleen, lymph node and thymus cells from primary (1°) doxycycline-dependent H9M-ciMP induced AMLs. (a-b) Immunophenotype analysis of spleen (Spl) and lymph node (LN) cells from DOX-treated 1° recipients transplanted with H9M-ciMPs cultured under myelomonocytic (36S) or stem cell maintaining (STIF) conditions. Flow cytometry plot overlays present expression of CD11b/Gr-1 and CD44/B220 in the EGFP$^+$dTomato$^+$ and EGFP$^-$dTomato$^+$ cells. **(c)** Flow cytometry plot overlays present expression of CD11b/Gr-1 and CD44/CD25 in the EGFP$^+$dTomato$^+$ and EGFP$^-$dTomato$^+$ thymus (Thy) cells. Frequency percentages are given for recipients #224 (36S) and #228 (STIF). AML, acute myeloid leukaemia; H9M-ciMPs, conditionally immortalised myeloid progenitor cell lines engineered to co-express *Hoxa9* and *Meis1*.

Supplementary Figure S8

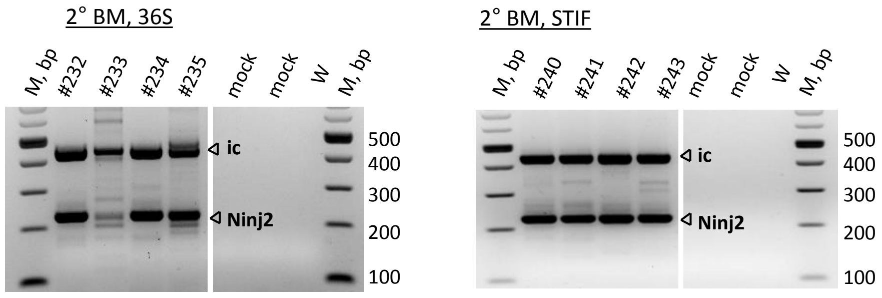

Supplementary Figure S8. Insertional analysis of secondary (2°) doxycycline-dependent H9M-ciMP induced AMLs. Ligation-mediated PCR analyses demonstrating insertion sites in bone marrow (BM) of 2° 36S and STIF recipients. W, water; M, 100 bp marker; bp, base pairs; ic, internal control; mock, BM cells from non-transplanted doxycycline-treated mice; H9M-ciMPs, conditionally immortalised myeloid progenitor cell lines engineered to co-express *Hoxa9* and *Meis1*; 36S, myelomonocytic conditions; STIF, stem cell maintaining conditions; AML, acute myeloid leukaemia.

Supplementary Figure S9

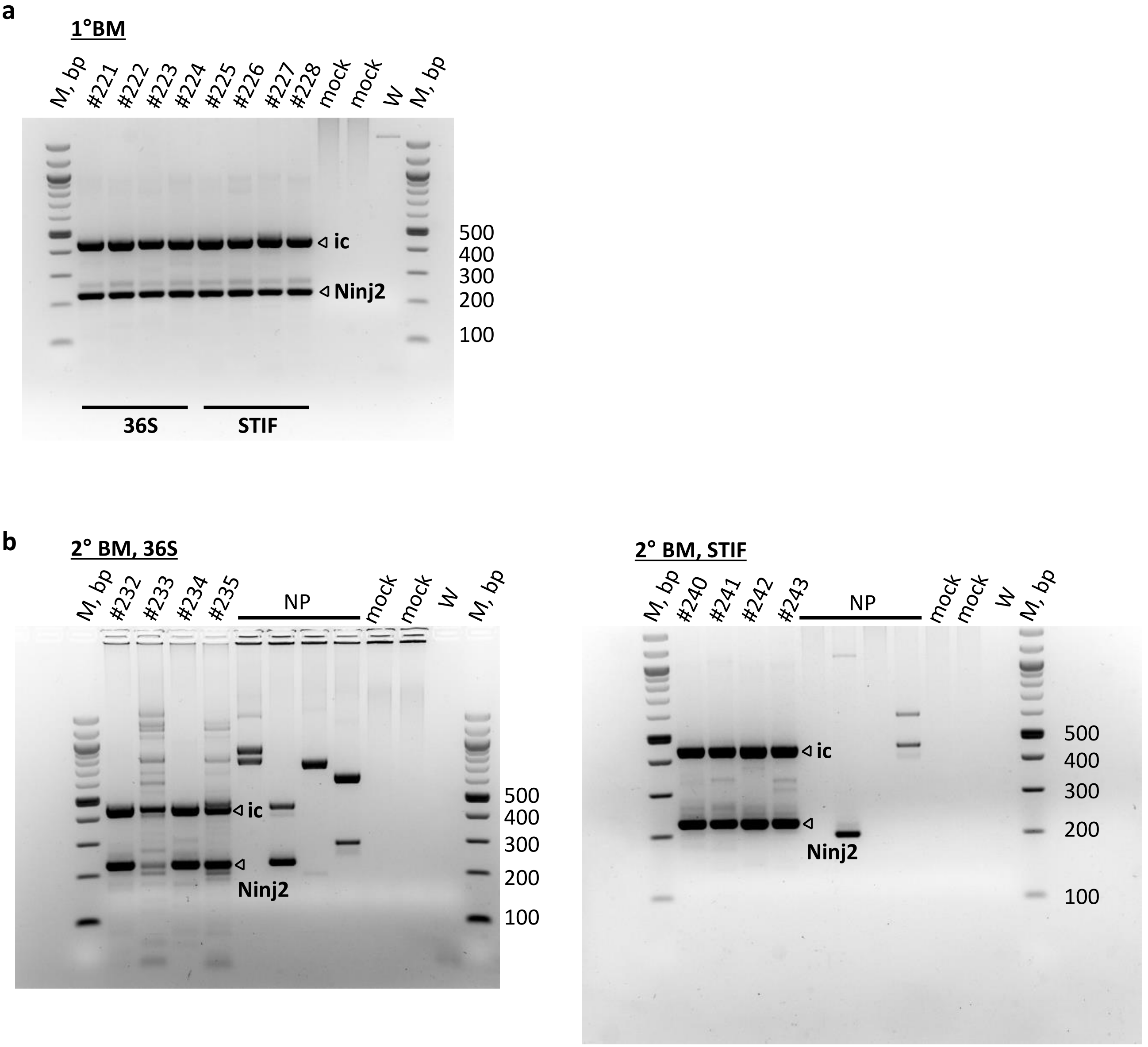

Supplementary Figure S9. The images of full-length gel-electrophoresis for Ligation-mediated PCR analyses. The pictures of full-length gels correspond to Figure 5e **(a)**, Supplementary Figure S8 **(b)**. 1°, primary recipients; 2°, secondary recipients; BM, bone marrow cells; W, water; M, 100 bp marker; bp, base pairs; ic, internal control; mock, BM cells from non-transplanted doxycycline-treated mice; H9M-ciMPs, conditionally immortalised myeloid progenitor cell lines engineered to co-express *Hoxa9* and *Meis1*; 36S, myelomonocytic conditions; STIF, stem cell maintaining conditions; NP, not presented.

Supplementary Figure S10

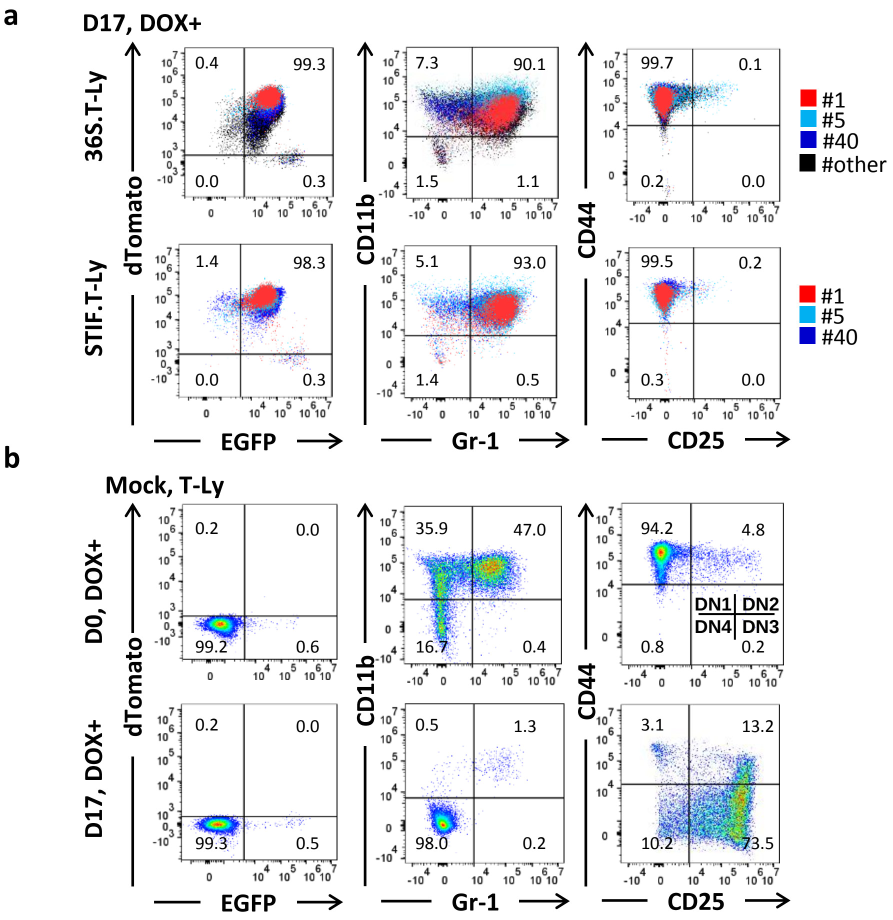

Supplementary Figure S10. Flow cytometric analysis of H9M-ciMPs cultured under T-lymphoid conditions *ex vivo*. (a) Dot plot overlays to present expression of EGFP/dTomato, CD11b/Gr-1 and CD44/CD25 (gated on viable cells) in H9M-ciMPs cultured under 36S.T-Ly and STIF.T-Ly conditions on day 17. H9M-ciMPs #1, 5 and 40 are marked in red, light blue and blue, respectively. Other H9M-ciMPs (#11, 12, 24, 25, 29) are marked in black. Frequency percentages are given for H9M-ciMP cell line #1. **(b)** Expression of EGFP/dTomato, CD11b/Gr-1 and CD44/CD25 markers (gated on viable cells) in lineage negative cells (Mock non-transduced controls) in co-culture with OP9-DL1 stromal cells under T-lymphoid (T-Ly) conditions on days 0 and 17 (cells were pre-cultured for 4 days under the T-Ly conditions). DN, double-negative stages. D, day; DOX+, 1 µg/mL doxycycline. 36S.T-Ly and STIF.T-Ly, co-culture with OP9-DL1 stromal cells under T-lymphoid cytokine conditions following 36S (myelomonocytic) and STIF (stem cell maintaining) conditions, respectively. H9M-ciMPs, conditionally immortalised myeloid progenitor cell lines engineered to co-express *Hoxa9* and *Meis1*.

Supplementary Figure S11

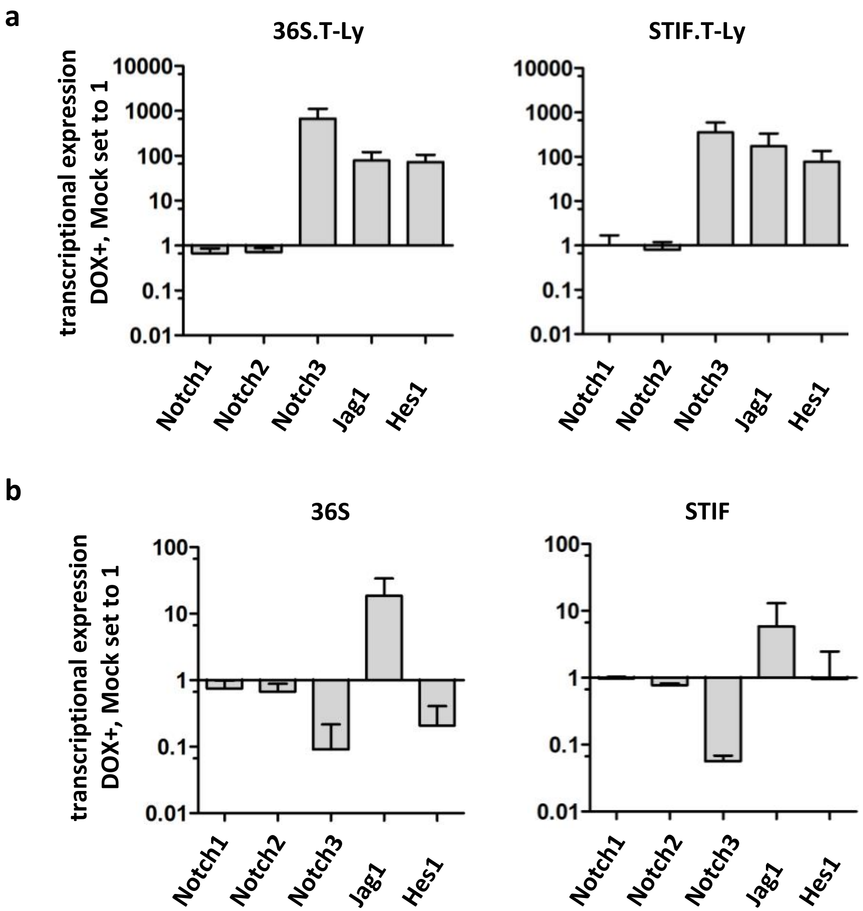

Supplementary Figure S11. Transcriptional dysregulation of Notch signalling genes in H9M-ciMPs cultured under different microenvironmental conditions *ex vivo* in the presence of doxycycline. Histogram presentation of relative transcriptional expression in H9M-ciMPs treated with 1 µg/mL of doxycycline under **(a)** T-lymphoid conditions 36S.T-Ly and STIF.T-Ly, **(b)** myelomonocytic (36S) and stem cell maintaining (STIF) conditions. The expression levels in non-transduced non-cultured lineage negative cells (Mock) were set to 1. Data are represented as mean ± SD, *n*=3 (36S.T-Ly, STIF.T-Ly, STIF), *n*=8 (36S). Levels of *Notch3* expression were compared for 36S.T-Ly *versus* 36S, STIF.T-Ly *versus* STIF; the differences are statistically significant, **, P<0.01. Levels of *Hes1* expression were compared for 36S.T-Ly *versus* 36S, STIF.T-Ly *versus* STIF; the differences are statistically significant, *, P<0.05. Comparisons were made using the non-parametric two-tailed Mann-Whitney test. 36S.T-Ly and STIF.T-Ly, co-culture with OP9-DL1 stromal cells under T-lymphoid cytokine conditions following 36S and STIF conditions, respectively. H9M-ciMPs, conditionally immortalised myeloid progenitor cell lines engineered to co-express *Hoxa9* and *Meis1.*

Supplementary Figure S12

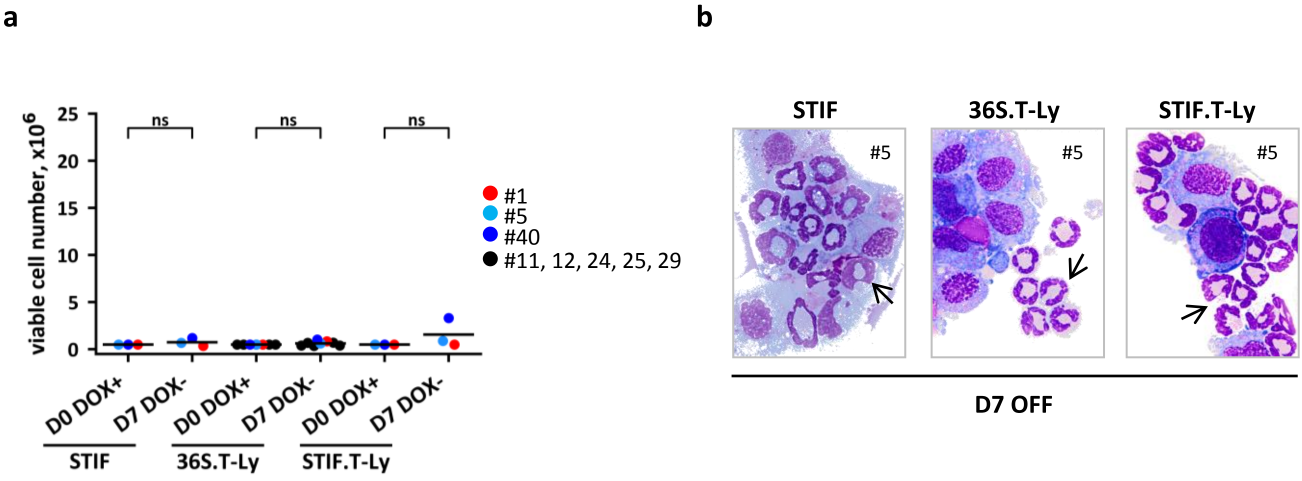

Supplementary Figure S12. Characterisation of H9M-ciMPs after doxycycline removal under different microenvironmental conditions *ex vivo*. (a) Viable cell number for H9M-ciMPs cultured under different microenvironmental conditions *ex vivo* after doxycycline (DOX) removal (D7 DOX-). STIF, stem cell maintaining (*n*=3); 36S.T-Ly (*n*=8) and STIF.T-Ly (*n*=3), co-culture with OP9-DL1 stromal cells under T-lymphoid conditions following 36S and STIF conditions, respectively. H9M-ciMPs #1,5,40 are marked in red, light blue and blue, respectively, and #11,12,24,25,29 are marked in black. Each data point represents the result of an individual cell line. Horizontal lines represent the mean values. ns, not significant, $p > 0.05$ (non-parametric two-tailed Mann-Whitney test). **(b)** Cytospin analysis of depicted H9M-ciMP #5 after DOX removal (D7 DOX-) under STIF, 36S.T-Ly and STIF.T-Ly conditions (May-Grünwald/Giemsa staining, magnification x100). Arrows indicate neutrophils. D, day. H9M-ciMPs, conditionally immortalised myeloid progenitor cell lines engineered to co-express *Hoxa9* and *Meis1*.

Supplementary Table S1. Generation of conditionally immortalised myeloid progenitor cell lines co-expressing *Hoxa9* and *Meis1* (H9M-ciMPs) and testing under different conditions *ex vivo* and *in vivo* under doxycycline supplementation.

Vector/ experiment	36S pre-selected → LM-PCR→ STIF tested H9M-ciMP, #	STIF survived H9M-ciMP, #	36S^x → 36S.T-Ly tested H9M-ciMP, #	36S.T-Ly survived H9M-ciMP, #	STIF^x → STIF.T-Ly tested H9M-ciMP, #	STIF.T-Ly survived; H9M-ciMP, #	Mouse, #	Donor cells: H9M-ciMP #, cytokine conditions; mouse #	No. of transplanted cells per mouse	No. of fresh BM competitor cells	No. of mice	DOX treatment before Tx	DOX treatment after Tx	*in vivo*, weeks
MRV, *ex vivo*, 1µg/mL DOX	1, 5	1, 5	1, 5	1, 5, 11, 12	1, 5	1, 5								
	9, 11, 12, 14, 15, 17, 24		11, 12		NA									
	22		NS		NA									
MRV*, *ex vivo*, 1µg/mL DOX	5,11,12,18	24, 25, 29, 40	NS	24, 25, 29, 40	NA	40								
	24, 25, 28, 29		24, 25, 29		NS									
	38, 40, 41, 42		40		40									
1° MRV, *in vivo*							221-224	1 + 5, 36S	$0.8x10^6$	$3x10^5$	4	1 µg/mL	625 mg/kg	8
							225-228	1 + 5, STIF	$0.7x10^6$	$3x10^5$	4	1 µg/mL	625 mg/kg	8
							229-231	mock	NA	NA	3	NA	625 mg/kg	8
2° MRV, *in vivo*							232-235	222, 36S	$1.8x10^6$	0	4	625 mg/kg	625 mg/kg	2
							240-243	225, STIF	$1.8x10^6$	0	4	625 mg/kg	625 mg/kg	2
							248-250	mock	NA	NA	3	NA	625 mg/kg	2

MRV, multimodal retroviral vector; MRV*, multimodal retroviral vector without adapter for LM-PCR; 36S, myelomonocytic cytokine conditions; STIF, stem cell maintaining cytokine conditions; 36S.T-Ly, T-lymphoid conditions following cultivation in 36S; STIF.T-Ly, T-lymphoid conditions following cultivation in STIF; 36S^x, technical replicates from 36S pre-selected H9M-ciMP; STIF^x, technical replicates from STIF survived H9M-ciMP; NS, not studied; NA, not applicable; mock, not transplanted mice; DOX, doxycycline; BM, bone marrow; Tx, transplantation; LM-PCR, ligation-mediated PCR; No., number; 1°, primary murine transplantation; 2°, secondary murine transplantation. For testing under different cytokine conditions: H9M-ciMPs from the same biological replicate are indicated by horizontal lines.

Supplementary Table S2. Insertional analysis of H9M-ciMP cell lines and 1° and 2° AMLs.

Hit, vector	H9M-ciMP, BMT recipient (#)	Chromosome	Locus	Gene ID (NCBI)	Official full name	Position to TSS, bp, (i)	Orientation
1, MRV	#1<br>#5<br>1°#221-224, BM<br>1°#225-228, BM<br>2°#232-235, BM<br>2°#240-243, BM	6 F1	Ninj2	29862	ninjurin 2	21914 (i1)	forward
			Wnk1	232341	WNK lysine deficient protein kinase 1	-76592	reverse
			B4galnt3	330406	beta-1,4-N-acetyl-galactosaminyl transferase 3	179295	reverse
			Rad52	19365	RAD52 homolog, DNA repair protein	212566	forward
			Ccdc77	67200	coiled-coil domain containing 77	249110	reverse
			Kdm5a	214899	lysine (K)-specific demethylase 5A	-248860	forward
2, MRV	#9<br>#11<br>#14<br>#15<br>#17<br>#24	3 F2.1	Vps45	22365	vacuolar protein sorting 45	-10346	reverse
			Otud7b	229603	OTU domain containing 7B	-35715	forward
			Plekho1	67220	pleckstrin homology domain containing, family O member 1	-72811	reverse
			Mtmr11	194126	myotubularin related protein 11	-93192	forward
			Sf3b4	107701	splicing factor 3b, subunit 4	-103520	forward
			Sv2a	64051	synaptic vesicle glycoprotein 2 a	-112339	forward
3, MRV	#12	9 A5.3	Plet1	76509	placenta expressed transcript 1	-10949	reverse
			Rpl10-ps3	100043346	ribosomal protein L10, pseudogene 3	-138608	forward
			Pts	19286	6-pyruvoyl-tetrahydropterin synthase	45148	forward
			Il18	16173	interleukin 18	-71251	reverse
			Bco2	170752	beta-carotene oxygenase 2	71605	forward
			Tex12	66654	testis expressed 12	77749	forward
4, MRV		17 E4	Thada	240174	thyroid adenoma associated	137390 (i28)	reverse
			Zfp36l2	12193	zinc finger protein 36, C3H type-like 2	-140868	reverse
			Plekhh2	213556	pleckstrin homology domain containing, family H (with MyTH4 domain) member 2	-183080	forward
			Dync2li1	213575	dynein cytoplasmic 2 light intermediate chain 1	-297681	forward
5, MRV	#22	13 C3	Xrcc4	108138	X-ray repair complementing defective repair in Chinese hamster cells 4	615 (i1)	reverse
			Tmem167	66074	transmembrane protein 167	-130	forward
			Vcan	13003	versican	-346484	reverse
6, MRV*	#5<br>#11	17 A3.2	Lnpep	240028	leucyl/cystinyl aminopeptidase	24293 (i1)	reverse
			Vmn2r90	626942	vomeronasal 2, receptor	-102990	forward

	#12				90		
			Lix1	66643	limb and CNS expressed 1	198085	forward
			Spaca6	75202	sperm acrosome associated 6	-226401	forward
			Has1	15116	hyaluronan synthase 1	254448	reverse
7, MRV*	#18	10 D2	4930471E19Rik	74912	RIKEN cDNA 4930471E19 gene	36626 (i1)	forward
			4921513I03Rik	70874	RIKEN cDNA 4921513I03 gene	-103155	reverse
			Hmga2	15364	high mobility group AT-hook 2	-186108	forward
			Msrb3	320183	methionine sulfoxide reductase B3	236524	forward
			1700006J14Rik	321010	RIKEN cDNA 1700006J14 gene	298420	reverse
8, MRV*	#24 #25 #28 #29	13 A3.1	Cmah	12763	cytidine monophospho-N-acetylneuraminic acid hydroxylase	79471 (i2)	forward
			Ripor2	193385	RHO family interacting cell polarization regulator 2	-94650	forward
			Carmil1	68732	capping protein regulator and myosin 1 linker 1	-126080	reverse
			Gmnn	57441	geminin	355048	reverse
9, MRV*	#40	7C	Chsy1	269941	chondroitin sulfate synthase 1	102494	forward
			Lrrk1	233328	leucine-rich repeat kinase 1	176341	reverse
			Aldh1a3	56847	aldehyde dehydrogenase family 1, subfamily A3	215508	reverse
			Selenos	109815	selenoprotein S	132360	forward
			Snrpa1	68981	small nuclear ribonucleoprotein polypeptide A'	153006	forward
10, MRV*	#41	13 A3.1	Ripor2	193385	RHO family interacting cell polarization regulator 2	140003 (i1)	reverse
			Gmnn	57441	geminin	120395	forward
			BC005537	79555	cDNA sequence BC005537	-160129	reverse
			Acot13	66834	acyl-CoA thioesterase 13	190012	forward
			Tdp2	56196	tyrosyl-DNA phosphodiesterase 2	-190151	reverse
			Cmah	12763	cytidine monophospho-N-acetylneuraminic acid hydroxylase	314124	reverse
11, MRV*	#42	12 E	Asb2	65256	ankyrin repeat and SOCS box-containing 2	15733 (i2)	forward
			Fam181a	100504156	family with sequence similarity 181, member A	29293	reverse
			Otub2	68149	OTU domain, ubiquitin aldehyde binding 2	-48414	reverse
			Ddx24	27225	DEAD box helicase 24	85562	forward
			Ifi27	52668	interferon, alpha-inducible protein 27	-93943	reverse
			Prima1	170952	proline rich membrane anchor 1	-98118	forward

Supplementary Table S2. Insertions sites revealed by LM-PCR in H9M-ciMP cell lines. H9M-ciMP, conditionally immortalised myeloid progenitor cell lines co-expressing *Hoxa9* and *Meis1*; underlined H9M-ciMPs were selected for further study; BMT, bone marrow transplantation; 1°, primary recipients; 2°, secondary recipients; AML, acute myeloid leukaemia; BM, bone marrow cells; bp, base pair; i, intron. Insertions are defined with respect to the transcriptional start sites (TSS, the mRNA start according to the Ensembl database, release 98 – August 2020) of neighbouring genes. Gene

ID refers to the NCBI database. MRV, multimodal retroviral vector with LM-PCR linker; MRV*, multimodal retroviral vector without LM-PCR linker; LM-PCR for H9M-ciMP #38 did not reveal any specific bands.
